# Engineered dendritic cells from cord blood and adult blood accelerate effector T cell immune reconstitution against HCMV

**DOI:** 10.1038/mtm.2014.60

**Published:** 2015-01-07

**Authors:** Anusara Daenthanasanmak, Gustavo Salguero, Bala Sai Sundarasetty, Claudia Waskow, Kadriye Nehir Cosgun, Carlos A Guzman, Peggy Riese, Laura Gerasch, Andreas Schneider, Alexandra Ingendoh, Martin Messerle, Ildar Gabaev, Benno Woelk, Eliana Ruggiero, Manfred Schmidt, Christof von Kalle, Constanca Figueiredo, Britta Eiz-Vesper, Constantin von Kaisenberg, Arnold Ganser, Renata Stripecke

**Affiliations:** 1 Department of Hematology, Hemostasis, Oncology and Stem Cell Transplantation, Hannover Medical School, Hannover, Germany; 2 Regeneration in Hematopoiesis, DFG-Center for Regenerative Therapies Dresden, Technische Universität Dresden, Dresden, Germany; 3 Department of Vaccinology and Applied Microbiology, Helmholtz Centre for Infections Research, Braunschweig, Germany; 4 Institute of Virology, Hannover Medical School, Hannover, Germany; 5 Department of Translational Oncology, National Center for Tumor Diseases and German Cancer Research Center, Heidelberg, Germany; 6 Department of Transfusion Medicine, Hannover Medical School, Hannover, Germany; 7 Department of Obstetrics and Gynecology, Hannover Medical School, Hannover, Germany

## Abstract

Human cytomegalovirus (HCMV) harmfully impacts survival after peripheral blood hematopoietic stem cell transplantation (PB-HSCT). Delayed immune reconstitution after cord blood (CB)-HSCT leads to even higher HCMV-related morbidity and mortality. Towards a feasible dendritic cell therapy to accelerate *de novo* immunity against HCMV, we validated a tricistronic integrase-defective lentiviral vector (coexpressing GM-CSF, IFN-α, and HCMV pp65 antigen) capable to directly induce self-differentiation of PB and CB monocytes into dendritic cells processing pp65 (“SmyleDCpp65”). *In vitro,* SmyleDCpp65 resisted HCMV infection, activated CD4^+^ and CD8^+^ T cells and expanded functional pp65-specific memory cytotoxic T lymphocytes (CTLs). CD34^+^ cells obtained from PB and CB were transplanted into irradiated NOD.Rag1^−/−^.IL2γc^−/−^ mice. Donor-derived SmyleDCpp65 administration after PB-HSCT stimulated peripheral immune effects: lymph node remodeling, expansion of polyclonal effector memory CD8^+^ T cells in blood, spleen and bone marrow, and pp65-reactive CTL and IgG responses. SmyleDCpp65 administration after CB-HSCT significantly stimulated thymopoiesis. Expanded frequencies of CD4^+^/CD8^+^ T cell precursors containing increased levels of T-cell receptor excision circles in thymus correlated with peripheral expansion of effector memory CTL responses against pp65. The comparative *in vivo* modeling for PB and CB-HSCT provided dynamic and spatial information regarding human T and B cell reconstitution. *In vivo* potency supports future clinical development of SmyleDCpp65.

## Introduction

Human cytomegalovirus (HCMV) reactivation is clinically documented in 60–80% of allogeneic hematopoietic stem cell transplantation (HSCT) recipients causing graft rejection and increasing morbidity and leukemia relapse-related mortality.^1,2^ HCMV reactivation and disease risks increase for the combination of seropositive recipients and seronegative donors, because donor-derived protective T cell immunity against HCMV cannot be adoptively transferred as donor-lymphocyte infusion during or after HSCT.^3^ Another scenario that delays the immune reconstitution against HCMV is the haploidentical transplantation of highly purified CD34^+^ stem cells in pediatric^4^ and adult HSCT recipients.^5^ Due to the profound *ex vivo* T cell depletion, the *de novo* expansion of the T cell repertoire after haploidentical CD34^+^ peripheral blood HSCT (PB-HSCT) may adopt the thymus-dependent pathway. Donor-derived bone marrow progenitor cells migrate to the thymus for positive selection of T cells with a functional T cell receptor (TCR) and negative selection of autoreactive T cells. The resulting naive T cells that leave the thymus (recent thymic emigrants (RTE)) repopulate the secondary lymphatic tissues. There, they can be optimally activated by professional antigen presenting cells (such as dendritic cells (DCs)) to generate memory and effector responses. In humans transplanted with CD34^+^ selected cells, the production of substantial numbers of new naive T cells by the thymus is usually detected by 100 days post-transplant.^6^ Therefore, approximately until 100 days post-HSCT, patients are at particularly high risk for HCMV infection or reactivations. Umbilical cord blood transplantation (CB-HSCT) offers several practical advantages: relative ease of procurement and feasibility of cryo-banking, the absence of risk for donors, the reduced likelihood of transmitting infections (such as HCMV), and lower stringency for HLA matching (up to two HLA disparities out of six for malignant disease are acceptable). Pre-emptive and intensive pharmacological strategies are undertaken to prevent HCMV, but nearly 100% of seropositive CB-HSCT patients reactivate HCMV early post-transplant.^7^ Although diverse polyclonal HCMV-specific T cell responses can be seen early (even at 42 days) in patients who undergo double CB-HSCT, it has been proposed that they fail to expand to sufficient numbers or immune efficacy to control virus.^8^ Therefore, novel cell immune therapy approaches to accelerate adaptive *de novo* reconstitution after haploidentical or CB transplantation are specially desired to control HCMV reactivation episodes early after HSCT.

DCs play a central role in lymphatic tissues that are key for immune synapses with T and B cells for stimulation of specific and lasting immunity.^9,10^ *Ex vivo* generation of monocyte-derived DC cultured with different combinations of cytokines leads to terminal differentiation of postmitotic and nonreplicating DC that resemble natural myeloid DC in expression of several immunologic markers and antigen-presentation functions *in vitro*. One major limitation of conventional monocyte-derived DC (moDC) tested extensively in cancer clinical trials were their poor viability and migration to lymph nodes after administration.^11^ Nevertheless, moDC pulsed with HCMV tegument phosphoprotein (pp65) peptides used to immunize 24 allogeneic PB-HSCT patients in phase 1/2 clinical trials showed development of detectable HCMV-specific CD4^+^ and CD8^+^ T cell responses in five patients, correlating with lowering HCMV viral loads.^12^ Due to manufacturing and logistic demands for fast production of donor-derived antigen-pulsed moDC, larger multicentric clinical trials remain a challenge. Use of moDC in CB-HSCT is even more difficult, because the number of starting monocytes in CB is very small.

In order to improve the viability and potency of DCs and to simplify their manufacturing, we developed “*S*elf-differentiated *my*eloid-derived *le*ntivirus-induced *DCs*” (SmyleDC/pp65).^13^ SmyleDC/pp65 were previously generated from CD14^+^ PB monocytes after a single-step overnight coinfection with a combination of a bicistronic integrase-defective lentiviral vector (ID-LV) coexpressing human granulocyte–macrophage colony stimulation factor (GM-CSF) plus interferon (IFN)-α and a monocistronic vectors expressing the full length pp65 protein. SmyleDC/pp65 produced after ID-LV cotransduction with two separate vectors generated potent *in vivo* expansion of adoptively transferred T cells in a nonconditioned NOD.Rag1^−/−^.IL2γc^−/−^ (NRG)/huPBL model.^13^

NOD-scid.IL2γc^−/−^ (NSG) and NRG mice are becoming preferred immune deficient mouse strains for “humanization”, particularly as models recapitulating human lympho-hematopoietic cell engraftment and immune reconstitution studies. PB-HSCT and CB-HSCT models have been developed for both strains, but NRG mice have the advantage of higher radioresistance.^14^ Studies performed with purified CD34^+^ cells obtained from CB and transplanted into irradiated NSG mice showed that after 16–22 weeks, thymus, spleen, and mesenteric lymph nodes (LN) were highly repopulated with human B and T cells, whereas peripheral LN in other parts of the body were undetectable.^14,15^ In agreement with a predominant naive human T cell phenotype observed in these tissues, the combinatorial diversity of the TCR-β chain generated in the thymus of CB-transplanted NSG was overall preserved in the periphery.^15^ These facts suggested that, although human T cells at least to some extent developed in the thymus, were not well activated in secondary lymphatic tissues to result into further amplification and functional memory T cell responses. Although the CB-HSCT humanized mouse models showed practical uses for human thymocyte development *in vivo*, the later stages of T cell development were vastly compromised making this a stringent model of human immune reconstitution.

In a previous study, we explored DC to enhance the activation/maturation of T cells generated in NRG after PB-HSCT.^16^ We compared the effects of SmyleDC/pp65 and moDC/pp65 immunizations, both expressing pp65 endogenously after ID-LV transduction. Remarkably, only SmyleDC/pp65, which were able to effectively migrate to the LN “Anlage” of the NRG mice promoted the regeneration of lymph nodes. This was associated with significant expansion of mature human T and B cells with specificity against pp65.^16^

In this study, we sought to further develop the generation of genetically reprogrammed DC for immunization of immune compromised hosts. In order to facilitate future production of lentivirus-reprogrammed DC with a single vector compliant with good manufacturing practices, we constructed a tricistronic LV expressing simultaneously the cytokines and pp65. We showed that transduction of monocytes obtained from PB and CB with this vector consistently generated within one day “SmyleDCpp65” that could be administered directly as an autonomously differentiating DC vaccine. We characterized the identity and potency of SmyleDCpp65 *in vitro* using HCMV infection and T cell stimulation assays. In addition, the two different relevant models recapitulating human PB-HSCT and CB-HSCT were used to evaluate the potency of SmyleDCpp65 *in vivo*. Both models demonstrated the *de novo* generation of human effector cytotoxic T lymphocytes (CTL) reactive against pp65. Interestingly, immunizations with SmyleDCpp65 after PB-HSCT showed a marked extra-thymic T cell development and IgG responses, like boost immunizations. On the other hand, SmyleDCpp65 immunizations after CB-HSCT resulted in significantly higher thymopoiesis but lower effects on secondary lymphatic tissues and no IgG production. These results reflect what is seen after primary immunization. These notable differences in the *in vivo* immune reconstitution of adult and neonate stem cells highlight the value of different humanized mouse models to evaluate distinct patterns of immune reconstitution after PB or CB-HSCT.

## Results

### SmyleDCpp65 produced with a tricistronic vector: viability and identity

**Figure 1:**
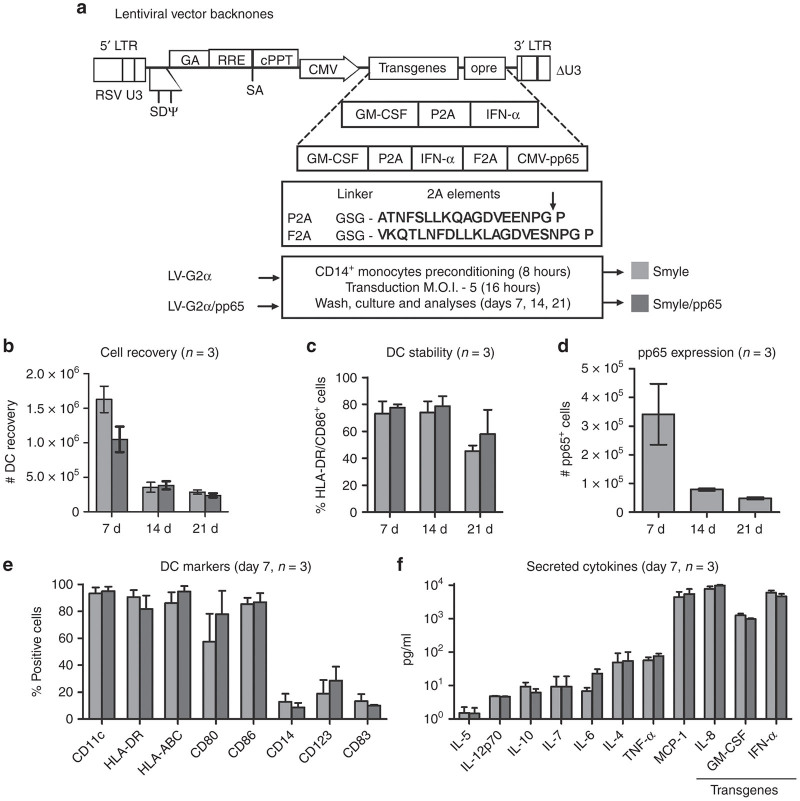
Generation and analyses of SmyleDC and SmyleDCpp65 from peripheral blood mononuclear cells of healthy donors. (**a**) Lentiviral vector backbones. Scheme of the chimeric multicistronic LV-G2α and LV-G2α-pp65 vectors. The open reading frames in the bicistronic LV vector encoded for GM-CSF and IFN-α and tricistronic lentiviral (LV) vector encoded for GM-CSF, IFN-α, and HCMV-pp65. Each gene was separated by a 2A element from the porcine teschovirus (P2A) upstream of human IFN-α and F2A (foot and mouth disease virus) upstream of HCMV-pp65. ID-LV-G2α and ID-LV-G2α-pp65 were used to generate SmyleDC and SmyleDCpp65 by transducing 5 × 10^6^ preconditioned monocytes (multiplicity of infection of 5) for 16 hours. The cells were washed, cultured, and analyzed on days 7, 14, and 21. (**b**) Cell recovery. Total viable SmyleDC (light grey) and SmyleDCpp65 (dark grey) recovered from DC cultures on days 7, 14, and 21 were determined as absolute number relative to input of transduced monocytes. (**c**) DC Stability. Flow cytometry analysis was used to determine frequency of cells that were double positive for CD86^+^/HLA-DR^+^ on days 7, 14, and 21. (**d**) Absolute numbers of cells positive for pp65 expression analyzed by intracellular staining on days 7, 14, and 21. (**e**) Expression of relevant DC immunophenotypic markers (CD11c, HLA-DR, HLA-ABC, CD80, CD86, CD14, CD123, and CD83) determined as percentages of positive cells on day 7. (**f**) Secreted cytokines detectable in SmyleDC and SmyleDCpp65 cultures. Supernatants were collected on day 7 (IL-4, IL-5, IL-6, IL-7, IL-8, IL-10, IL-12p70, MCP-1, TNF-α) and transgenic cytokines (GM-CSF and IFN-α) were measured using cytokine bead arrays. All analyses were performed as independent triplicates with monocytes obtained from three different donors.

The tricistronic self-inactivating ID-LV-G2α-pp65 was designed with heterologous interspaced 2A elements (P2A derived from porcine teschovirus and F2A from foot and mouth disease virus) (Figure 1a). Large-scale batches of third generation ID-LV pseudotyped with the vesicular stomatitis virus—G protein (VSV-G) were produced as previously described.^13^ Viral titers were determined by measuring concentration of the p24 capsid protein, resulting in titers in the normal range (tricistronic: 7 µg/ml, *n* = 10; bicistronic: 9.4 µg/ml, *n* = 10) (Supplementary Information, Figure S1a). The expression of all the transgenes GM-CSF, IFN-α, and pp65 in transduced 293T cells were confirmed by analyses of lysates and cell supernatants by western blot and immune detection. GM-CSF and IFN-α proteins were primarily secreted and detectable in the cell supernatants, whereas the pp65 protein remained intracellular (Supplementary Information, Figure S1b,c). Overnight exposure of monocytes obtained from healthy donors (HD) with the ID-LV-G2α or ID-LV-G2α-pp65 vectors at a multiplicity of infection of 5 and subsequent *ex vivo* culture in the absence of recombinant cytokines resulted in similar DC induction (Figure 1b). Monocytes isolated from blood from G-CSF PB donors or CB were also induced to DC with the ID-LV-G2α-pp65 vector (Supplementary Information, Figure S1d,e). Recovery of CD14^+^ monocytes of the total Peripheral blood mononuclear cells (PBMC) in the CB units and of SmyleDCpp65 of the monocytes used for transduction were, in average, 11.3 and 45.3% of the input cells, respectively (*n* = 3, Supplementary Information, Table S1). Stability of immunophenotypic markers (HLA-DR^+^/CD86^+^) and detectable levels of intracellular pp65 expression in SmyleDCpp65 of monocytes obtained from PB were analyzed for 21 days of *ex vivo* culture period (Figure 1c,d), as after this time point the cells lost viability. Detailed analyses on day 7 of both DC cultures showed high frequencies of CD11c^+^, HLA-DR^+^, HLA-ABC^+^, CD80^+^, CD86^+^ cells, and low frequencies of monocytes (<10% CD14^+^), plasmacytoid DCs (< 40% CD123^+^) and terminally differentiated DCs (<10% CD83^+^) (Figure 1e). SmyleDCpp65 secreted several cytokines (low to moderate levels up to 10 pg/ml: IL-5, IL-12p, IL-10, IL-7, IL-6, IL-4, and TNF-α; high levels 1–10 ng/ml: IL-8 and MCP-1) and high levels of the transgenic GM-CSF and IFN-α cytokines (~1.0 and 4.6 ng/ml, respectively (*n* = 3)) (Figure 1f). In conclusion, we observed no adverse effects of pp65 coexpressed *in cis* in the tricistronic vector.

### SmyleDCpp65 resists to HCMV replication *in vitro*

**Figure 2:**
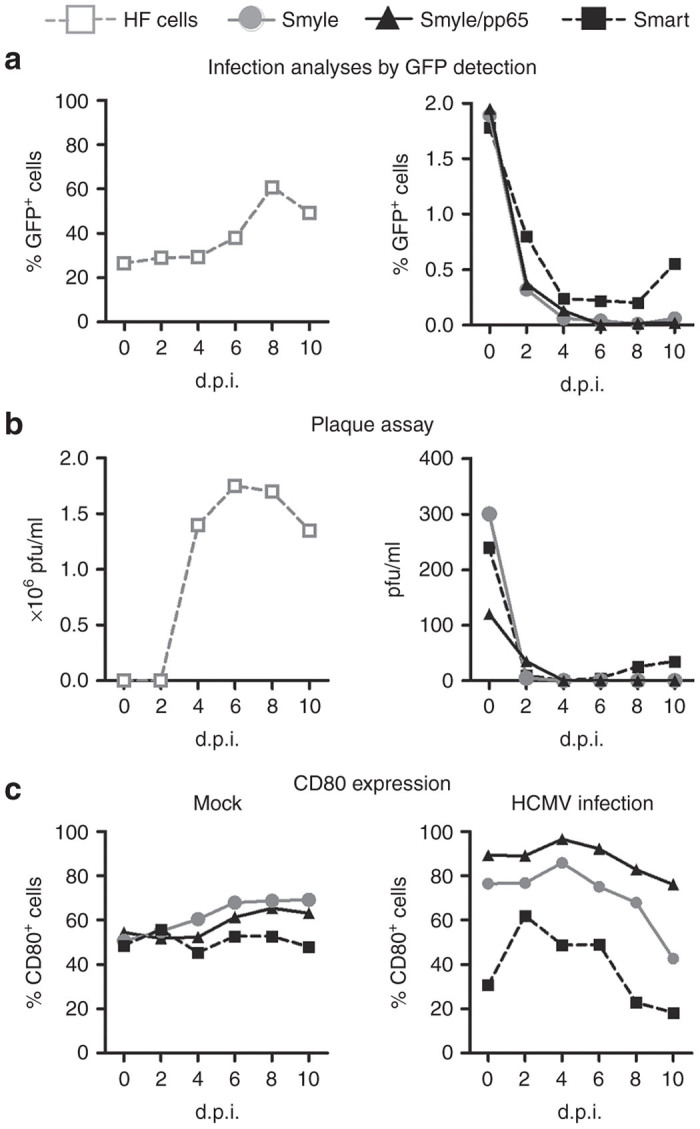
Infection of dendritic cells (DCs) with human cytomegalovirus (HCMV) *in vitro*. (**a**) Flow cytometry analyses for detection of HCMV/GFP^+^ infected cells at 0–10 day postinfection (d.p.i.) control human fibroblast (HF) cells (gray square, left graph) and different types of lentivirus-induced DC; SmyleDC (grey circle), SmyleDCpp65 (dark triangle), and SmartDC (dark square). Multiplicity of infection of 1 was used in the assay. (**b**) Detection of newly released HCMV virions in cell supernatants. Supernatants from each infected cell culture were collected on 0, 2, 4, 6, 8, and 10 d.p.i and evaluated for the presence of infectious particles by plaque assay. Control infected HF cells supernatants (left graph) and DC supernatants (right graph) are shown. Titers are represented as particle forming units (pfu)/ml. (**c**) Flow cytometry analyses of CD80^+^ cells comparing noninfected DCs (mock) with HCMV-infected DCs showing dynamic changes in CD80 expression on different d.p.i.

MoDCs are known to be susceptible to HCMV infection and, upon their differentiation into activated DCs, virus replication can be observed.^17^ Therefore, one important safety aspect of SmyleDCpp65 was whether they would support HCMV spread. To address this, different types of lentivirus-induced DCs were compared: SmyleDC coexpressing GM-CSF and IFN-α with SmartDC coexpressing GM-CSF and IL-4.^13^ DCs were infected with the genetically modified viral strain HCMV-TB40/E expressing GFP at multiplicity of infection of 1. Infected human fibroblasts (HF) were used as positive controls as previously described^18^ (Figure 2a). Ten days after infection, ~50% of the HF cells were GFP-positive cells by flow cytometry analysis. Approximately 2.0% of the DC cultures showed initial HCMV uptake. For SmartDC, the frequency of GFP^+^ cells decreased initially, but then bounced back to 0.6% on day 10. For SmyleDC and SmyleDCpp65, the frequencies of GFP^+^ cells were lower than 0.06% (Figure 2a). To evaluate whether infected cells could release new virions, supernatants collected from each time points were analyzed by plaque assay (Figure 2b). Infected HF showed high amounts of virus release from day 4 to 10 (>1.4 × 10^6^ pfu/ml) and most of the cells of the culture became lysed. Infectious viruses were detectable on day 0 of DC cultures (120–300 pfu/ml), possibly reflecting remaining carry-over virus sticking on DC surface (Figure 2b). SmartDC started to release virus on day 6 (5 pfu/ml), which gradually increased on day 10 (to 35 pfu/ml). In contrast, SmyleDC or SmyleDCpp65 did not release virus. Thus, IFN-α expression by SmyleDC and SmyleDCpp65 controlled HCMV infection and release, whereas IL-4 expression by SmartDC did not. These analyses were complemented by monitoring CD80, a relevant costimulatory molecule shown to be modulated after HCMV infection.^19^ The frequency of CD80^+^ cells was constant in noninfected mock cells (50%), whereas it declined sharply in HCMV-infected SmartDC. Remarkably, for both types of SmyleDC, the frequency of CD80^+^ cells initially increased upon HCMV infection (80%), being maintained at constant level in SmyleDCpp65 for 10 days (Figure 2c).

### SmyleDCpp65 stimulate and expand autologous pp65-reactive CTLs *in vitro*

**Figure 3:**
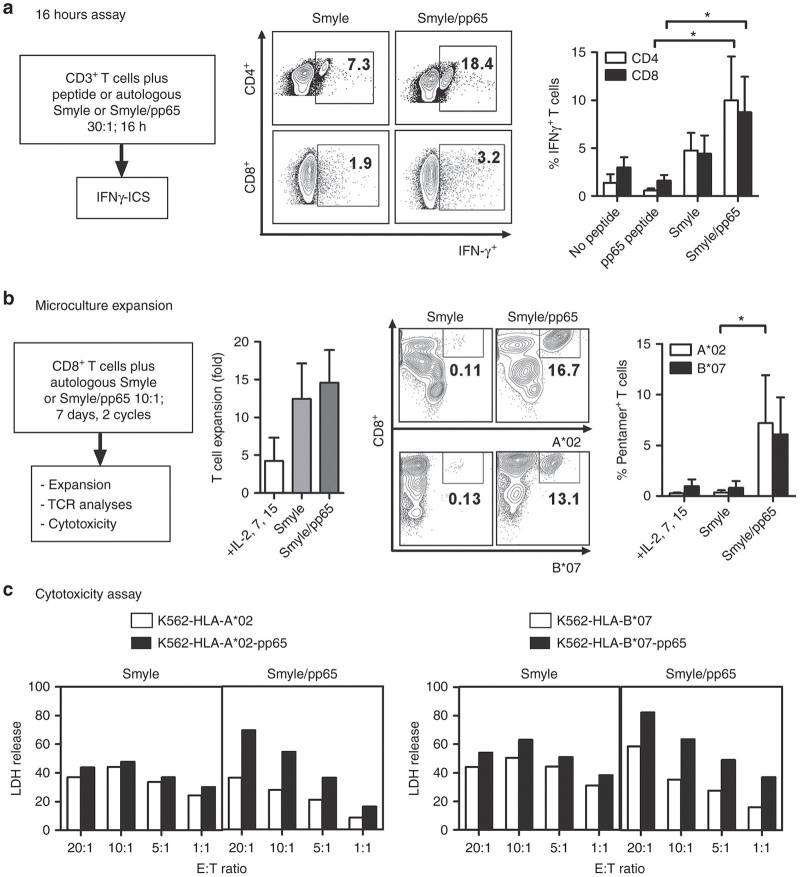
Stimulation of T cell with SmyleDC and SmyleDCpp65 *in vitro*. (**a**) Short stimulation of T cells measured by interferon (IFN)-γ ICS. CD3^+^ T cells obtained from HCMV seropositive donors (*n* = 3) were either not stimulated, stimulated with pp65 peptide, with SmyleDC or with SmyleDCpp65 *in vitro* for 16 hours at T cell to DC ratio of 30:1. Representative CD4^+^/IFN-γ^+^ and CD8^+^/IFN-γ^+^ FACS analyses from one donor determined by IFN-γ intracellular staining are shown as a density plot. Bar graph: Frequencies of IFN-γ^+^ producing CD4^+^ (white) and CD8^+^ (black) T cells were calculated from triplicate independent experiments. Student *t*-test was used for calculation of *P <* 0.05. (**b**) Long stimulation of CD8^+^ T cell expansion in T cell microculture. Selected CD8^+^ T cells were stimulated with SmyleDC or SmyleDCpp65 *in vitro* at T cell and DC ratio of 10:1 for two weekly cycles in the presence of irradiated autologous feeder cells plus recombinant IL-2, IL-7, and IL-15 cytokines. T cells stimulated with feeders and cytokines were used as controls. Left bar graph: viable T cells stimulated with either cytokine and feeder cells, SmyleDC or SmyleDCpp65 were counted and plotted as fold expansion relative to input (1 × 10^6^ cells). pp65 specificity measured by pentamer staining. Expanded T cells were stained with pentamers reactive against pp65 epitopes. Representative pp65-restricted CD8^+^/A*0201^+^ and CD8^+^/B*0702^+^ analyses of T cells expanded from one donor determined by flow cytometry analyses. Right bar graph: frequencies of T cells restricted to A*0201-NLVPMVATA: white/B*0702-TPRVTGGGAM: black. Student *t*-test was used for calculation of *P* < *0.05*. (**c**) Cytotoxic activity of CD8^+^ expanded T cells cocultured with K562 target cells controls or expressing pp65 epitopes and presented through HLA -A*02 or B*07 context (at different effector: target (E:T) ratios (20, 10, 5, and 1:1) for 4 hours. LDH release measured by coupled enzymatic assay was used to quantify K562 target lysis. T cells homeostatically expanded with SmyleDC were cocultured with K562 cells (−/+ expressing pp65) were used as cytotoxicity controls.

Both CD4^+^ T helper1 (Th1) cells and CD8^+^ CTLs are required to control HCMV replication during reactivation after HSCT.^20–22^ A 16-hour IFN-γ catch assay based on flow cytometry analysis of cryopreserved/thawed selected CD3^+^ T cells was used to evaluate whether SmyleDCpp65 (harvested on day 7 after transduction) could activate both types of T cells obtained from HCMV seropositive HD (*n* = 3) (Figure 3a). As controls, we included no stimulation, stimulation with pp65 peptide pool (the standard positive control for this assay) and SmyleDC not coexpressing pp65 antigen. Under the assay conditions using selected CD3^+^ T cells in the absence of feeder cells or other antigen presenting cells, we did not observe an increase in the frequency of IFN-γ producing CD4^+^ or CD8^+^ cells upon stimulation with pp65 peptides (Supplementary Figure S2). Notably, SmyleDC not loaded or expressing pp65 antigens stimulated CD4^+^ and CD8^+^ T cells. The lentiviral vector expressing GM-CSF and IFN-α does not express any additional protein (the pre-element was mutated and a protein is not expressed). Thus, this activation is likely due to homeostatic signals. Combining SmyleDC and exogenous loading with the pp65 peptide pool considerably increased CD4^+^ and CD8^+^ T cell activation. Surprisingly, when pp65 peptides were used to load SmyleDCpp65, we saw an effect of CD4^+^ T cells, but no additional effects on CD8^+^ T cell activation. This indicated that the endogenous pp65 processing and presentation provided more potent signals to CD8^+^ T cells than the exogenous peptide loading. Experiments performed in triplicates to specifically compare the effects of SmyleDC versus SmyleDCpp65 are shown in Figure 3a. Stimulation of CD3^+^ T cells with SmyleDCpp65 resulted in significant increases in the frequency of IFN-γ producing CD4^+^ T cells (18-fold, *P <* 0.05) and CD8^+^ T cells (fivefold, *P* < 0.05). SmyleDC not loaded with pp65 antigen promoted a weaker induction of IFN-γ production in CD4^+^ and CD8^+^ cells, again, likely due to homeostatic effects of IFN-α on activation of T cells for production of IFN-γ.^23^ To further evaluate the direct effects of SmyleDCpp65 in the activation of pp65-specific CD8^+^ memory T cells, we performed two sequential microcultures of DCs and T cells obtained from HLA A*02; B*07 donors (*n* = 3, HCMV seropositive) in order to expand CTLs (Figure 3b). Coculture of purified CD8^+^ T cells with autologous SmyleDC or SmyleDCpp65 both resulted in comparable T cell expansion (13-fold), indicating that this effect was partly due to homeostatic cytokine effects. Nevertheless, pentamer analyses for detection of pp65-specific TCRs showed that only SmyleDCpp65 stimulated expansion of pp65-specific T cells (A*02 restricted epitope pp65 aa 495–503: mean = 7.7%, *P* < 0.05; B*07 restricted epitope pp65 aa 417–426: mean 6.4%, *P* < 0.1) (Figure 3b). CTLs expanded in the presence of SmyleDC or SmyleDCpp65 were subsequently evaluated for cytotoxic function. We used K562 cell targets genetically modified for constitutive expression of HLA-A*02 (KA*02) or HLA-B*07 (KB*07), which were further modified for constitutive pp65 expression (Figure 3c). CTLs expanded with SmyleDC showed similar cytotoxicity activity upon coincubation with KA*02 or KB*07 targets, regardless if the pp65 antigen was expressed in the target or not. In contrast, CTLs stimulated with SmyleDCpp65 in a dose-dependent manner more effectively lysed K562 target cells expressing pp65 (Figure 3c). This data validated the effects of SmyleDCpp65 to stimulate and expand pp65-specific CTLs *in vitro*.

### Accelerated hematopoietic reconstitution of NRG mice after human PB-HSCT and adoptive transfer of SmyleDCpp65

**Figure 4:**
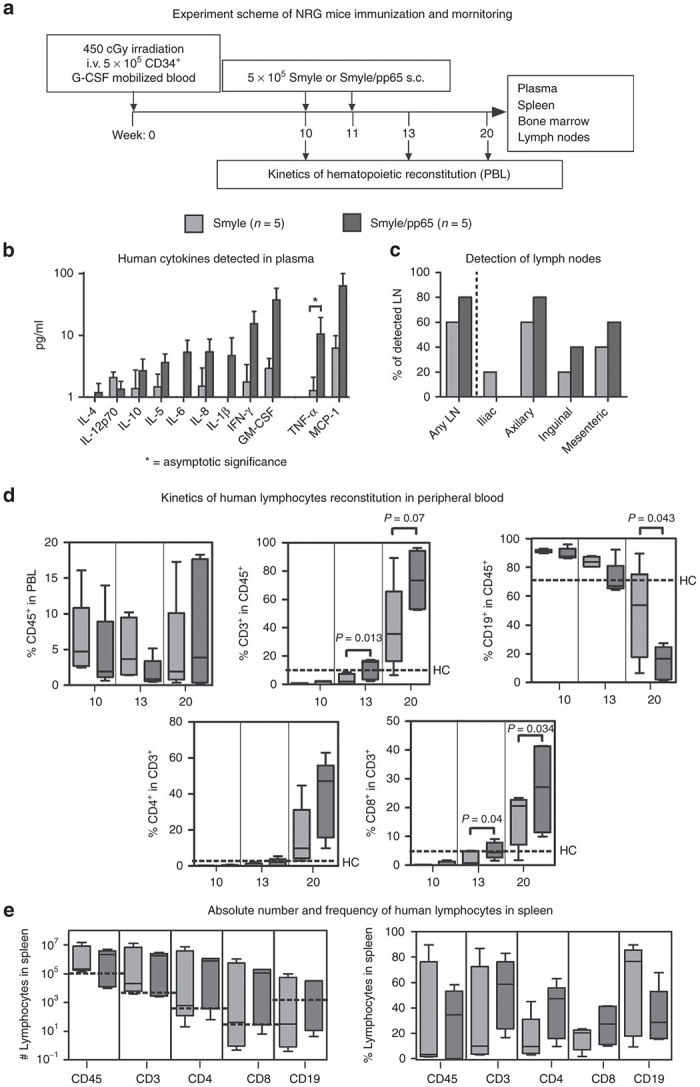
NRG mice transplanted with adult CD34^+^ HSC and immunized with SmyleDC or SmyleDCpp65. (**a**) Experimental scheme of immunization and monitoring. Four-week-old irradiated NRG mice (450 cGy) were transplanted with 5 × 10^5^ PB CD34^+^ stem cells isolated from G-CSF mobilized blood. On weeks 10 and 11 after hematopoietic stem cell transplantation (HSCT), mice were immunized subcutaneously on the hind flanks with either 5 × 10^5^ SmyleDC or SmyleDCpp65. Kinetics of human hematopoietic reconstitution in blood was determined by flow cytometry at week 10 (before immunization) and week 13 (after immunization). Mice were sacrificed and blood, plasma, spleen, bone marrow and lymph nodes were collected 20 weeks after transplantation. For all analyses, SmyleDC (light grey, *n* = 5) and SmyleDCpp65 (dark grey, *n* = 5) were compared. (**b**) Bar graphs showing concentrations of human cytokines measured by cytokine bead arrays detected in mice plasma (pg/ml) IL-4, IL-12p70, IL-10, IL-5, IL-6, IL-8, IL-1β, TNF-α, IFN-γ, GM-CSF, and MCP-1 were. * represents asymptotic significance analyzed by Kolmogorov–Smirnov test. (**c**) Detection of lymph nodes. Frequency as percentage of mice in the cohort with detectable LN at any location or at different body locations for each group. (**d**) Kinetics of human lymphocytes reconstitution in PB determined by flow cytometry. Frequencies of human CD45^+^, CD3^+^ in CD45^+^, CD4^+^ in CD45^+^, CD8^+^ in CD45^+^, and CD19^+^ in CD45^+^ were determined on weeks 10, 13, and 20. Black dashed lines represent observed levels of human cells in historical PB-HSCT control group (no immunization, 20 weeks after HSCT). Box plot indicating median and error bars indicating ranges are shown and statistical analyses were determined by *post hoc* test; *P* < *0.05* was considered significant. (**e**) Box plot indicating median and error bars indicating ranges of absolute counts (right) and frequencies (left) of human lymphocytes detected in spleen; CD45^+^, CD3^+^ in CD45^+^, CD4^+^ in CD45^+^, CD8^+^ in CD45^+^, and CD19^+^ in CD45^+^ cells determined by flow cytometry.

NRG mice were transplanted with CD34^+^ cells obtained from G-CSF-mobilized stem cell donors (Supplementary Information, Table S1) and SmyleDC or SmyleDCpp65 administered on weeks 10 and 11 post-HSCT to evaluate the effects of pp65 expression on the immune reconstitution (Figure 4a). Mice were sacrificed 20 weeks after HSCT and, as expected based on our previous work,^16^ several human cytokines were detectable in the plasma (Supplementary Table S3). A strong trend for higher levels of inflammatory cytokines was observed after SmyleDCpp65 administration (IL-4, IL-10, IL-5, IL-6, IL-8, and in particular high levels IFN-γ, GM-CSF, TNF-α, and MCP-1) (Figure 4b) (Supplementary Information, Table S2a). The distinct upregulated expression of TNF-α and MCP-1 is remarkable, as both these cytokines have proangiogenic and tissue remodeling properties. Also, in accordance with our previous work,^16^ reconstituted lymph nodes containing human lymphocytes were readily detectable in various body locations, and higher frequencies of LN (at any location or at the different locations) was observed in 80% of the mice administered with SmyleDCpp65 (Figure 4c) (Supplementary Information, Figure S2a). This is an important observation, since NRG mice lack all murine lymphocytes (T, B, and NK cells) and do not develop LN at all. Therefore, the observed LN regeneration must result from immune reconstitution of human T and B cells, which are able to find and home in the murine LN “Anlage”. At baseline prior to SmyleDC or SmyleDCpp65 administration, analyses of the kinetics of hematopoietic reconstitution showed similar frequencies of huCD45^+^ cells in PBL (5–10%) (Figure 4d). This was comparable to our historical PB-HSCT controls not administered with DCs.^16^ Within the huCD45^+^ population, very low frequencies of CD3^+^ T cells could be initially observed (less than 5%), and most of the cells corresponded to CD19^+^ B cells (90%). The high frequency of human CD19^+^ B cells after HSCT in immune deficient mice does not parallel the clinical scenario, where T cells predominate. The pattern of low CD3^+^ T cells and high CD19^+^ B cell frequency changed drastically after SmyleDCpp65 administration and final analyses on week 20. We observed an increase in the T cell frequency (*P =* 0.07) and significant reduction of B cell frequency (*P* = 0.043). Frequency of CD4^+^ T cells and particularly CD8^+^ T cells (*P* = 0.034) increased after SmyleDCpp65 administration (Figure 4d). These effects were reproducible in the analyses of splenocytes, both in terms of absolute cells counts and relative cell frequencies (Figure 4e). Of note, in the cohort of SmyleDCpp65 administered mice, we observed that one of the mice developed xenograft graft versus host disease (xenoGVHD). This mouse was excluded from the immune reconstitution analyses as it showed a massive expansion of CD4^+^ memory T cells in blood, spleen, and bone marrow (Supplementary Information, Figure S3b,c). All the other mice did not show signs of GVHD, and it is possible that residual memory T cells from the HCMV seropositive HSC donor contaminating the SmyleDCpp65 preparation engrafted and expanded, causing xenograft GVHD effects.

**Figure 5:**
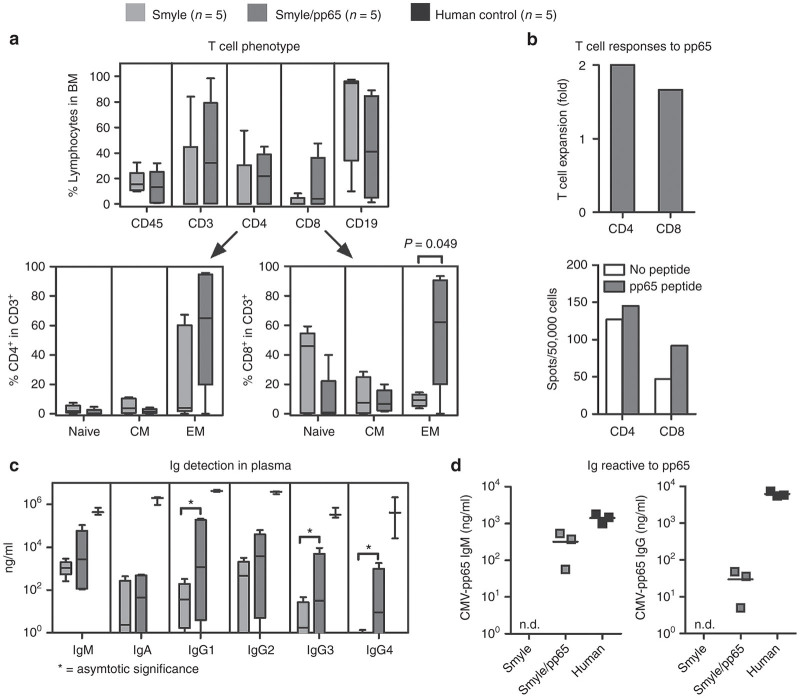
Functional effects of SmyleDCpp65 immunization after PB-HSCT. All Box plots indicate median and error bars indicate ranges of analyses performed 20 weeks after HSCT, SmyleDC (light grey, *n* = 5), and SmyleDCpp65 (dark grey, *n* = 5) were compared. (**a**) Upper panel: frequency of human lymphocytes and T cell subsets in bone marrow; CD45^+^, CD3^+^ in CD45^+^, CD4^+^ in CD45^+^, CD8^+^ in CD45^+^, and CD19^+^ in CD45^+^. Lower panels: subsets of T cells in CD4^+^ and CD8^+^ populations classified as naive, central memory (CM), and effector memory (EM). Analysis of variance with *post hoc* analysis test was used to determined significance, *P* < 0.05. (**b**) T cell responses against pp65. CD4^+^ or CD8^+^ T cells were sorted from splenocytes explanted from SmyleDCpp65-immunized NRG mice (*n* = 3) and pooled. T cells were homeostatically activated with CD2/CD3/CD28 beads for 48 hours followed by 7 days *ex vivo* expansion with SmyleDCpp65. 50,000 T cells were restimulated overnight with either HCMV-pp65 overlapping peptide pool or without peptide on anti-IFN-γ-coated ELISPOT plates. Bars represent number of IFN-γ-positive spots. (**c**) Human immunoglobulin (Ig) levels of IgM, IgA, IgG1, IgG2, IgG3, and IgG4 (ng/ml) detectable by bead arrays assay in plasma of NRG mice immunized with SmyleDC (*n* = 5) or SmyleDCpp65 (*n* = 5) in comparison with plasma obtained from human donors (*n* = 3). (**d**) Anti-pp65 reactivity of human IgM and IgG in plasma of mice immunized with SmyleDC or SmyleDCpp65 measured by ELISA. Human HCMV seropositive donors were used as positive controls (*n* = 3).

Analyses of phenotypic T cells markers in bone marrow of mice administered with SmyleDCpp65 showed comparable frequencies of CD4^+^ and CD8^+^ T cells, with a predominant effector memory (EM) phenotype. Conversely, mice receiving SmyleDC showed higher frequency of naive CD8^+^ T cells, but very few EM (Figure 5a). This demonstrated the strong effect of the pp65 antigenic stimulus in directing the T cell development towards terminal activation *in vivo*. In order to confirm anti-pp65-specific T cell responses, splenocytes of mice immunized with SmyleDCpp65 were sorted and pooled. Selected CD4^+^ and CD8^+^ T cells could be modestly activated and expanded *in vitro* to allow further analyses. After pulsing the expanded cells with a pp65 peptide mix, we observed CD8^+^ T cell reactivity, measured as quantified IFN-γ-positive spots (Figure 5b). Another parameter of functional immune reconstitution was Ig seroconversion analysis. Despite the broad variable responses in the cohort of mice analyzed, several types of human IgGs were detectable at higher levels after SmyleDCpp65 immunization (IgG1, IgG2, IgG3, and IgG4) (Figure 5c) (Supplementary Information, Table S2b). We could detect low levels of IgM and IgG reactivity against pp65 protein by ELISA, although much lower than human plasma obtained from HCMV seropositive donors (Figure 5d). Therefore, pp65 antigen presentation by SmyleDCpp65 could also promote Ig-switch during B cell development. This is a probable result of B cell activation by crosstalk effector pp65-reactive CD4^+^ T helper cells (Supplementary Information, Figure S4). Altogether, the PB-HSCT model showed a predominantly skewed EM T cell phenotype. The thymuses in these mice were small, underdeveloped, and scarcely repopulated with human cells, and therefore, the T cell development was extrathymic (Supplementary Information, Figure S4).

### Higher thymic T cell development, T cell output, and functional T cell responses in NRG mice after human CB-HSCT and SmyleDCpp65 immunization

**Figure 6:**
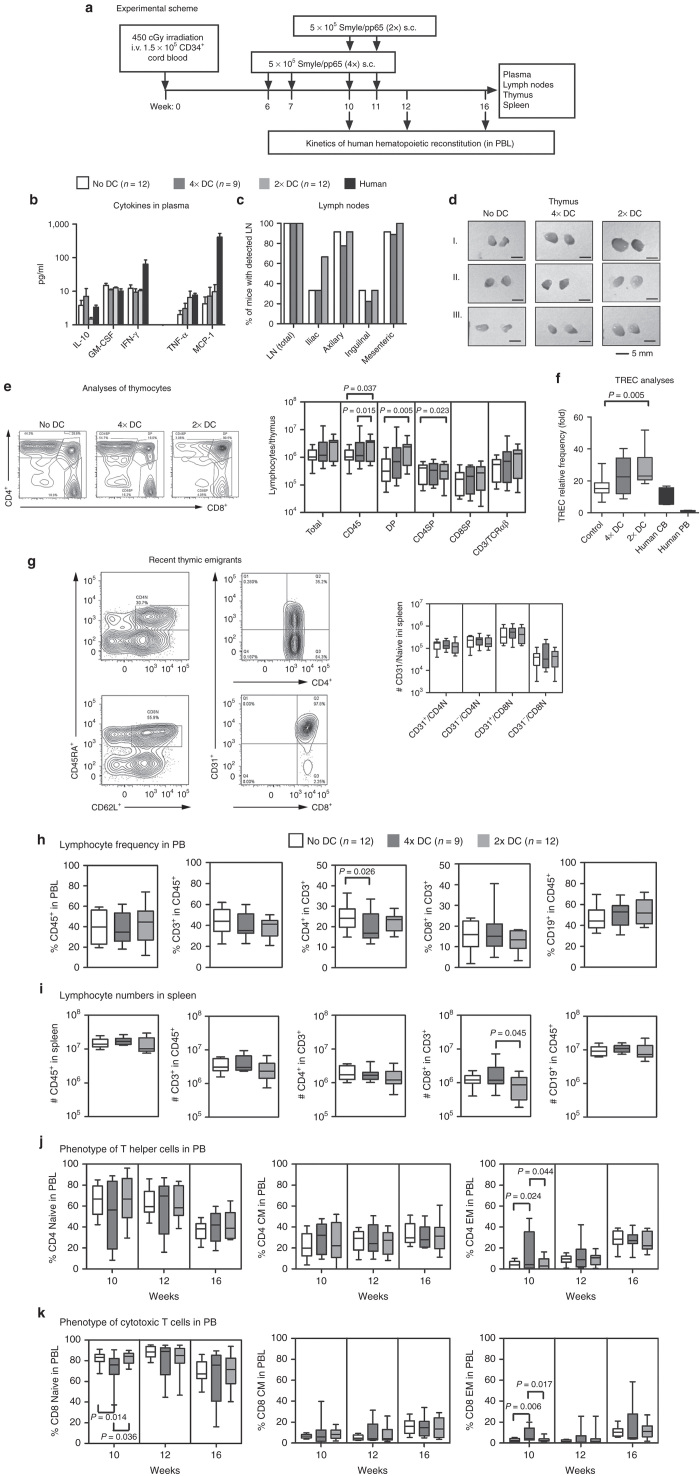
Mice transplanted with CB CD34^+^ HSC and immunized with SmyleDCpp65. (**a**) Scheme of immunization and monitoring. Four-week-old irradiated NRG mice transplanted with 1.5 × 10^5^ CB CD34^+^ cells immunized with SmyleDCpp65 four times on weeks 6, 7, 10, and 11 (dark grey, *n* = 9) or two times on weeks 10 and 11 (light gray, *n* = 12). Nonimmunized mice (no DC, white, *n* = 12) were used as controls. Kinetics of hematopoietic reconstitution in blood was determined by flow cytometry on weeks 10 and 12. Mice were sacrificed and blood, plasma, spleen, and thymus were collected 16 weeks after transplantation. All box plots indicate median and error bars indicate ranges of analyses. (**b**) Human cytokines detected in mice plasma. Level of cytokines (pg/ml) IL-10, IFN-γ, GM-CSF, TNF-α, and MCP-1 was measured by cytokine bead arrays. (**c**) Detection of lymph nodes. Frequency as percentage of mice in the cohort with detectable LN at any location or at different body locations for each group. (**d**) Macroscopic examination of thymus. Enlarged thymus was observed in SmyleDCpp65-immunized mice. (**e**) Left panels show representative CD4^+^/CD8^+^ gating strategy. Absolute cell counts in thymus. Analyses of thymocytes at different developmental stages; DP; CD45^+^/CD4^+^/CD8^+^, CD4SP; CD45^+^/CD4^+^/CD8^-^, CD8SP; CD45^+^/CD4^-^/CD8^+^, CD3/TCRαβ; CD45^+^/CD3^hi^/TCRαβ^+^. (**f**) T cell receptor excision circles (TREC) analysis of thymocytes by PCR. Frequency of TRECs in blood T cells from healthy donors (45–60 years) was set to 1 to calculate the relative frequency of TRECs in CB T cells and thymocytes from HSC-NRG mice. TREC value from human CB and PB were used as controls. Analysis of variance with *post hoc* test was used for statistical analyses; *P* < 0.05 considered significant. (**g**) Upper panels show representative gating strategy for detection of CD31^+^ thymic CD4^+^ and CD8^+^ naive T cell. Lower panel shows absolute cell counts in spleen of CD31^+^ thymic and CD31^−^ central CD4^+^ and CD8^+^ naive T cells. (**h**) Frequency of lymphocytes in blood 16 weeks after HSCT; CD45^+^, CD3^+^ in CD45^+^, CD4^+^ in CD45^+^, CD8^+^ in CD45^+^, and CD19^+^ in CD45^+^. (**i**) Absolute counts of human lymphocytes in spleen 16 weeks after HSCT; CD45^+^, CD3^+^ in CD45^+^, CD4^+^ in CD45^+^, CD8^+^ in CD45^+^, and CD19^+^ in CD45. (**j**) Analyses of CD4^+^ subsets and (**k**) analyses of CD8^+^ subsets in blood on weeks 10, 12, and 16 after HSCT determined as frequencies of naive (N), CM, and EM T cells.

CB is a rich source of HSC and progenitor cells at very immature stages of differentiation. Therefore, we explored the CB-HSCT as a more stringent model to address the effects of induced DCs in the hematopoietic reconstitution *in vivo*. CD34^+^ CB transplantation into NRG mice could be reproducibly established with a dose of 1.5 × 10^5^ cells injected i.v. into irradiated 4-week-old mice (Figure 6a) (Supplementary Information, Table S1). We compared nonimmunized mice with mice immunized with autologous SmyleDCpp65 using the same dose (5 × 10^5^) and schedule as previously performed for the PB-HSCT model, *i.e.*, at 10 and 11 weeks after HSCT (“2x DC”). In addition, taking into account the more immature HSC status and possible more anergic environment after CB-HSCT, we administered an extra earlier prime-boost SmyleDCpp65 immunization, on weeks 6 and 7 after HSCT (“4x DC”). Analyses of human cytokines in plasma of mice sacrificed 16 weeks after CB-HSCT revealed detectable levels of IL-10, GM-CSF, and IFN-γ, but without clear distinction among the treatment groups. Interestingly, reproducing the observations of the PB-HSCT model, the levels of TNF-α and MCP-1 in plasma were also higher for SmyleDCpp65 immunized mice (Figure 6b). For CB-HSCT, however, some small lymph nodes, particularly mesenteric and axillary, could be observed in similar frequencies in non-immunized and in SmyleDCpp65 immunized mice (Figure 6c). The most notable macroscopic difference was the increased size of the thymus in mice administered with SmyleDCpp65 (Figure 6d). In view of these results, we examined the early development of T cells in the thymus. Remarkably, we observed significantly higher huCD45^+^ cellularity and increased numbers of double positive (DP) CD4^+^/CD8^+^ and CD4^+^ single-positive (SP) T cells in mice receiving the later stage “2x” SmyleDCpp65 prime-boost (Figure 6e). The later stage “2x” prime/boost with showed significantly higher turn-over thymic T cell development than the “4x” immunization schedule initiated at an earlier time point. Thymocytes isolated from “2x” SmyleDCpp65 contained significantly higher numbers of TCR excision circles (TRECs) than control mice (for the “4x” cohort was also increased, but not significantly) (Figure 6f). Analysis of CD31 (PECAM-1) can be used to differentiate CD31^+^ thymic naive and CD31^−^ central naive CD4^+^ T cells in the peripheral blood of healthy humans^24^ and therefore CD31 expression has been proposed as a signature of RTE cells.^25^ Analyses of naive CD4^+^ T cells in spleen showed similar total numbers of CD31^+^ thymic naive and CD31^−^ central naive CD4^+^ T cells. In contrast, most of the naive CD8^+^ T cells in spleen corresponded to CD31^+^ thymic although CD8^+^ T cells obtained from “4x” SmyleDCpp65 showed a trend towards higher numbers of CD31^−^ central naive CD8^+^ T cells (Figure 6g). Analyses of the frequency of the different lymphocyte types in blood and absolute counts of human lymphocytes in spleen at 16 weeks after HSCT showed a modest increase in the CD8^+^ T cell population for the 4× immunization group (Figure 6h,i). Strikingly, phenotypic analyses of T cells during the immune reconstitution showed that at 10 weeks post-HSCT (just 3 weeks after the first prime boost) of the “4x” cohort, highly significant increases in the frequencies of the EM CD4^+^ and CD8^+^ T cells could be detected in peripheral blood. This indicates a fast immune stimulation effect produced by SmyleDCpp65 (Figure 6j,k). Conversely, the frequency of naive CD8^+^ T cells in blood showed a significant drop in the “4x” cohort immediately after immunization (week 10), and this normalized at later analyses times. The “2x” cohort, which received only the delayed SmyleDCpp65 immunization, did not show these effects even at later time-points. This demonstrated that earlier and more intense SmyleDCpp65 immunization did produce an effect on CTL reconstitution.

**Figure 7:**
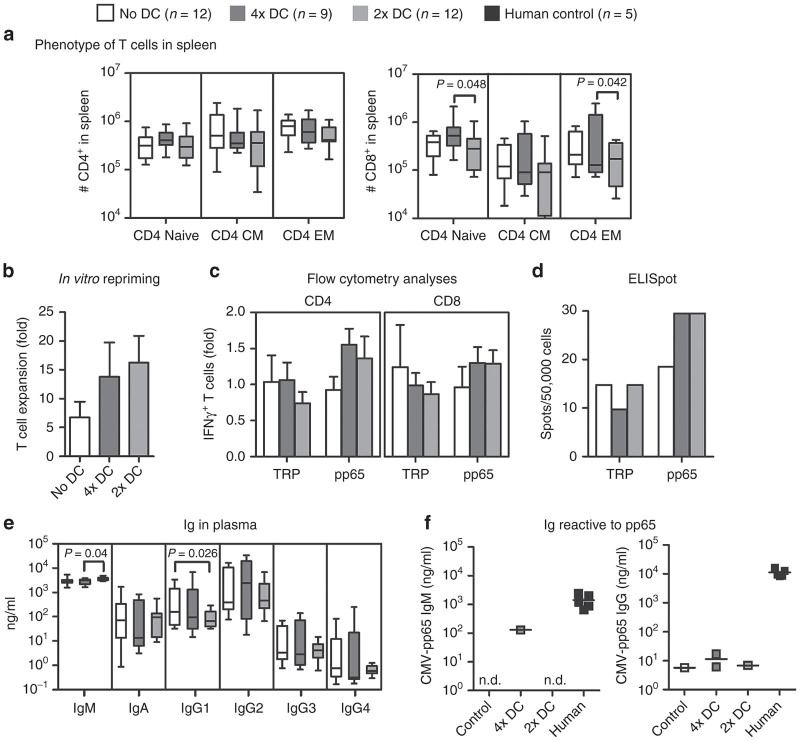
Functional effects of SmyleDCpp65 after CB-HSCT. All box plots indicate median and error bars indicate ranges of analyses performed 16 weeks after hematopoietic stem cell transplantation. (**a**) Absolute counts of CD4^+^ and CD8^+^ T cell subsets naive, CM, and EMT cells in spleen. Analysis of variance with *post hoc* test was used for statistical analyses; *P* < 0.05 considered significant. (**b**) T cell expansion in microculture. T cells explanted from lymph nodes (*n* = 4) were homeostatically activated with CD2/CD3/CD28 beads for 48 hours prior to *ex vivo* stimulation with SmyleDCpp65 for 7 days. Fold expansion was determined by number of expanded T cells on day 7 relative to cell input. (**c**) T cell responses against pp65 measured with interferon (IFN)-γ intracellular staining. Harvested T cells were either not stimulated with peptides (baseline control) or restimulated overnight *in vitro* in 96-wells plate with a pp65 overlapping peptide pool and a nonrelevant tyrosinase related protein (TRP)-2 peptide pool prior to IFN-γ ICS. Fold production was determined by % of IFN-γ^+^ T cells detected in peptide pulsed group/% of IFN-γ^+^ T cells from nonstimulated group. (**d**) ELISPOT assay (*n* = 2). 50,000 T cells were restimulated overnight with either human cytomegalovirus (HCMV)-pp65 overlapping peptide pool or TRP2 peptide pool on anti-IFN-γ-coated ELISPOT plate. Bars represent average numbers of IFN-γ-positive spots. (**e**) Human immunoglobulins (ng/ml); IgM, IgA, IgG1, IgG2, IgG3, and IgG4 detectable by bead arrays assay in plasma of NRG mice. (**f**) Anti-pp65 reactivity of human IgM and IgG in plasma of mice was measured by ELISA. Serum from HCMV seropositive donors were used as positive controls (*n* = 5).

The picture in spleen after 4× SmyleDCpp65 immunization also showed a significant increase in the absolute numbers of CD8^+^ EM T cells compared with 2× (Figure 7a). Interestingly, the absolute number of naive T cells was also augmented, indicating that emigrating naive CD8^+^ T cells from thymus reached the secondary lymphatic tissues, where they could further expand and differentiate into memory cells. T cells isolated from lymph nodes were reprimed *in vitro* with SmyleDCpp65. A much higher expansion was observed for T cells recovered from tissues of mice after CB-HSCT than PB-HSCT (~10-fold). The T cells were analyzed for reactivity against pp65 peptides by IFN-γ intracellular staining (Figure 7b,c) and ELISPOT (Figure 7d). These independent assays showed higher reactivity of T cells (both CD4^+^ and CD8^+^) expanded from SmyleDCpp65 mice (receiving “2x” or “4x” immunizations) against a pp65 peptide pool compared with nonimmunized control mice. Thus, although in absolute numbers the “2x” cohort seemed to be in general delayed in the T cell reconstitution compared with “4x”, their lymphocytes were also functionally reactive against pp65. Whereas the T cell compartment was clearly stimulated with SmyleDCpp65, the immunoglobulin analyses in the plasma showed a more complex situation. Mice immunized “4x” with SmyleDCpp65 showed a trend for higher IgG2 production, but mice immunized “2x” showed only increased IgM production. The levels of all the IgGs subtypes decreased for the “2x” cohort (Figure 7e). Only one out of nine of the mice immunized “4x” with SmyleDCpp65 showed detectable levels of pp65-specific IgM. IgGs reactive against pp65 were not detectable or at background levels (Figure 7f). The frequencies of CD4^+^FoxP3^+^CD25^+^CD127^-^ regulatory T cells (Tregs) in blood after SmyleDCpp65 administration was augmented compared with control mice (*P* = 0.06; data not shown) and none of the mice transplanted with CB developed GVHD. Altogether, the CB-HSCT model showed T cell development in thymus and expansion in secondary lymphatic tissues (Supplementary Information, Figure S4).

### Analyses of diversity of the TCR repertoire by sequencing

**Figure 8:**
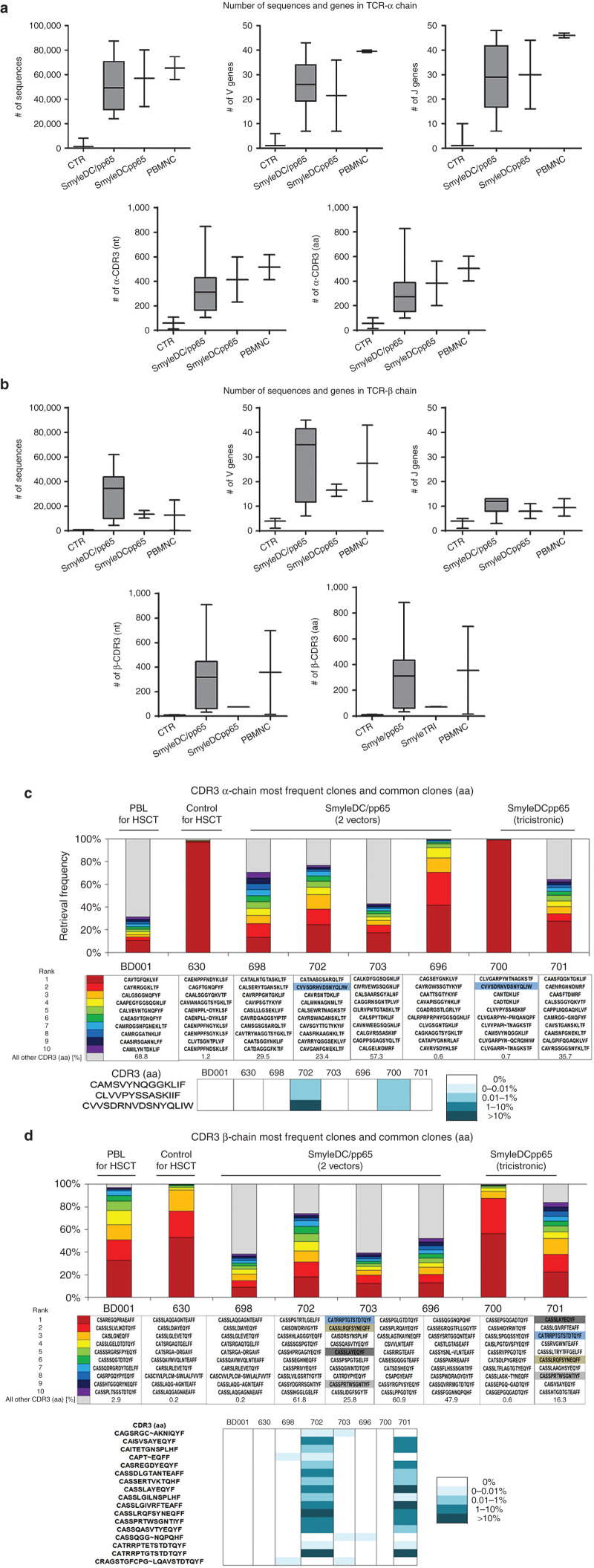
Analyses of T cell polyclonality by sequencing of genes encoding for the T cell receptor (TCR)-α and -β chains. (**a,b**) Individual plots represent number of sequences V genes, J genes, CDR3-nt clonotypes, and CDR3-aa clonotypes obtained from splenocytes of humanized mice (controls, *n* = 2; immunized with SmyleDC/pp65 generated with two vectors, *n* = 9 or with SmyleDCpp65 generated with the tricistronic vector, *n* = 2) in comparison with the PBMNC of stem cell donors (*n* = 2). (**c,d**) Upper panels: Graphic representation and sequence of the ten most frequent retrieved CDR3 sequences for TCR-α and -β chains. From red to violet the first and the tenth most predominant sequence are represented, grey indicates the remaining sequences. Larger grey areas represent higher polyclonality of a sample. Lower panel: detectable shared CDR3-aa sequences among different humanized mice that were immunized with SmyleDC/pp65 or SmyleDCpp65. aa, amino acid; CDR3, complementarity determining region-3; J, joining; nt, nucleotide; V, variable.

TCRs are dimeric (αβ of γδ) highly polymorphic proteins able to recognizing through the hypervariable complementarity determining region 3 (CDR3) antigenic peptides bound on MHC molecules. CDR3 region is originated by somatic rearrangement of noncontiguous variable (V), diversity (D, in β and δ chain) and joining (J) segments; additional random addition and deletion of nucleotides at the junction of the V(D)J recombinant gene further increase the TCR diversity. In order to evaluate the diversity of the α/β TCRs resultant from T cell development *in vivo* in humanized mice immunized with SmyleDCpp65, RNA encompassing the CDR3 region was amplified from mouse splenocytes by polymerase chain reaction (PCR) and sequenced. As positive controls, we included PBMNC samples obtained from CD34^+^ stem cell donors and monocytes. As additional experimental controls, we also included material obtained from humanized mice that were immunized with induced DCs generated by transduction with two different vectors (SmyleDC/ pp65) and described elsewhere.^16^ The main goal was to evaluate if TCR diversity in control, nonimmunized mice was qualitatively and quantitatively different from the TCR diversity found in mice immunized with induced DCs. We were able to include in the analyses data of nonimmunized mice (control, *n* = 2), immunized with SmyleDC/pp65 (*n* = 9) and SmyleDCpp65 (*n* = 2) (Supplementary Tables S4 and S5). For the α-chain, the PBMNC-positive control showed the highest output for total number of sequences (>60,000), number of V (~40) and J genes (~45), and diversity of the CDR3 region for nucleotide and amino acid sequence (~500 different sequences) (Figure 8a). T cells in control mice were polyclonal or oligoclonal (*i.e.*, less than 20 different clones were detectable) and the TCR diversity was dramatically inferior than PBMNC samples. For immunized mice, the TCR diversity was lower than PBMNC, but polyclonal (*i.e.*, more than 20 different clones were detectable). High TCR diversity for the β-chain was notably high for the PBMNC control and for the SmyleDC/pp65 immunized group, and still polyclonal for SmyleDCpp65 immunized mice (Figure 8b). In order to access the T cell repertoire in mice reconstituted with genetically identical human T cell progenitors, we further restricted the analyses to mice transplanted with CD34^+^ cells obtained from donor BD001. We plotted the relative frequency of the 10 dominant clones detected by analyses of the α and β chains (Figure 8c,d, respectively). Recapitulating the results obtained for the overall analyses, analyses of BD001 PBMNC control and resulting transplanted mice immunized with SmyleDC/pp65 yielded the highest diversity. Nevertheless, adopting the retrieval frequency of the top 10 sequences as parameters, we observed the presence of high dominant clones, corresponding to 30% to close to 100% of the sequences. One of the mice immunized with SmyleDCpp65 showed a polyclonal repertoire, whereas the repertoire of the second mouse was oligoclonal (similar to the nonimmunized control), which also reflected the lower number of sequences retrieved. Despite the small numbers of animals analyzed, we were able to deepen the analyses to look for the presence of shared CDR3 specificities in different humanized mice. For TCR-α, we were able to detect three sequences that were identical between two mice immunized with SmyleDC/ pp65 and SmyleDCpp65 (Figure 8c,d). For TCR-β, there was a much higher frequency of these shared CDR3 specificities (17 detectable from our analyses), occurring in several mice immunized with SmyleDC/pp65 and/or SmyleDCpp65, but none in the PBMNC control or nonimmunized group. Altogether, it seems that immunization of congenic humanized mice with immunogenic DCs could evoke the development and expansion of polyclonal T cells, and some antigen-experienced T cells could show identical CDR3 specificities.

## Discussion

Transplantation of allogeneic HSC is a validated therapeutic option for patients with high-risk hematological malignancies and hematopoietic failure syndromes, but HCMV infections pose a significant risk on overall survival and also is correlated with higher risk of leukemia relapse.^2^ HCMV causes significant morbidity after CB-HSCT, particularly to HCMV-seropositive patients. Immune reconstitution after CB-HSCT is quite delayed, and control of virus results mostly from T cells primed early after transplant.^8^ There is no currently marketed vaccine against HCMV. Several HCMV vaccine candidates including a live attenuated vaccine based on the Towne strain have been investigated and demonstrated partial efficacy in clinical trials.^26^ More recent attempts are based on recombinant proteins admixed with adjuvants^27^ and a DNA vaccine.^28^ Although immunogenic in immune competent mice and healthy volunteering individuals,^29^ these vaccine candidates may ultimately be suboptimal to induce productive immune responses and promote virus control in T lymphopenic patients after PB-HSCT or CB-HSCT. Expansion of viral-specific T cells *ex vivo* is an interesting alternative as further discussed below, but it requires several weeks and, remarkably, the resulting T cells seem to display a different preference for epitope specificity than observed in “natural” lymphocytes expanded in lymphatic tissues *in vivo*.^30^ The tegument pp65 is one of the candidate antigens for a HCMV vaccine, because a substantial fraction of CD8^+^ T cells of CMV-seropositive individuals is directed against epitopes of this viral protein.^31,32^ During the late phase of the HCMV infection cycle, pp65 is highly expressed in infected cells^33^ and is the most abundant protein in HCMV virions.^34^

In view of these facts, a personalized DC product directly stimulating immunity against HCMV-pp65 in lymphopenic host that could be easily produced in the clinical setting would be quite desirable. We had previously reported that NRG mice administered with SmyleDCpp65 and infused with adoptive donor lymphocytes from the same HCMV seropositive donor significantly accelerated T cell expansion and biodistribution, but characteristically resulted into fulminant GVHD.^13^ Recently, we demonstrated that NRG mice transplanted with CD34^+^ HSC obtained from G-CSF-mobilized donors and immunized with SmyleDCpp65 (produced with two vectors) at 10 and 11 weeks after HSCT showed 10 weeks later a significant acceleration of the human CD4^+^ and CD8^+^ effector memory T cell expansion, which was associated with the regeneration of lymph nodes and terminal differentiation of B cells.^16^ Detectable CTL and IgG specific against pp65 were detectable, demonstrating a full humanization of the adaptive immune responses in these mice.^16^

In this work, we confirmed the effect of SmyleDCpp65 produced with a single preclinical tricistronic ID-LV in generating these adaptive “switches” after PB-HSCT and CB-HSCT. Our results show that the tricistronic ID-LV encoding GM-CSF, IFN-α and the HCMV pp65 antigen under the control of the early CMV promoter can efficiently reprogram human CD14^+^ monocytes from PB and CB. A single overnight exposure of monocytes to the ID-LV vector drives their self-differentiation into DCs that are maintained autonomously for 2–3 weeks with high immunologic properties. Although it has been previously reported that ectopic expression of pp65 could inhibit IFN signaling,^35–37^ we did not observe a detrimental effect of pp65 coexpressed *in cis* in DCs. In fact, experimental infection of SmyleDCpp65 with HCMV *in vitro* showed that the cells resisted to HCMV, did not spread virus, and were refractory to immune downregulation. Clinically, pp65-specific functional CD4^+^ and CD8^+^ T cells were demonstrated to play a critical role in HCMV clearance. From our results, we observed a clear effect of endogenously expressed pp65 in the induced DC for stimulation of CD4^+^ and CD8^+^ T cell responses *in vitro*.

T cell immune reconstitution in humans following lymphodepletion can occur through active thymopoiesis or through homeostatic proliferation of peripherally expanded clones. Thymic development is slower, whereas homeostatic proliferation results in a rapid and significant expansion of the peripheral T cell (memory) pool, and is dependent upon both homeostatic cytokines and antigen-driven responses in the period following lymphocytopenia.^38^ The PB-HSCT humanized mouse model showed that the human T cell development occurred extrathymically. This pathway of T cell development may be rather not quite relevant for healthy individuals, but it seems to be of importance for individuals with a poor thymic function. Although the steps for extrathymic positive and negative human T cell selection are not fully elucidated, TCR rearrangements have been also detected within the human tonsil.^39^ Remarkably, the presentation of the immune dominant pp65 antigen by SmyleDCpp65 was essential in the PB-HSCT model for effective *de novo* peripheral T cell terminal differentiation. The CB-HSCT humanized mouse model recapitulated the slower human immune reconstitution and thymic T cell development preceded functional responses in lymphatic tissues. Overall, SmyleDCpp65 stimulated consistently higher frequencies of human effector CTLs in blood and spleen for both PB-HSCT and CB-HSCT. Noteworthy, lack of xenoGVHD despite the fact that active and polyclonal active human T cells are observed deserves further future studies. We speculate that SmyleDCpp65 directly or indirectly (through paracrine activation of other human DCs *in vivo*), can stimulate DC-dependent tolerogenic functions on the mouse thymic epithelial cells for negative selection of human T cells with TCRs highly reactive against mouse antigens. Alternatively, Treg induction could also be operative to suppress xenoGVHD. Convincingly, we provide evidence *in vivo* that SmyleDCpp65 can accelerate polyclonal homeostatic and antigen-specific T cell immune reconstitution after HSCT. In addition, one major limitation in immune reconstitution after human HSCT and also of humanized models of HSCT is the poor reconstitution of mature B cells and lack of Ig class switching. Remarkably, IgM, IgG and IgA were detected at high levels in PB-HSCT mice immunized with SmyleDCpp65, supporting the concept that pp65 antigen may strongly activate a T-helper dependent pathway leading to a helper-dependent maturation of B cells for Ig class switching. Studies in humanized mice demonstrated that B cell maturation was correlated with development of effector T cells.^40^ Thus, expansion of pp65-specific CD4^+^ T cells driven by SmyleDCpp65 may provide critical maturation factors such as CD40L that are required for B cell maturation, Ig class switch and secretion of antigen-specific antibodies.

Therefore, our studies comparing other differential effects of DC immunization after PB-HSCT or CB-HSCT, showed different patterns of T and B cell development: thymic or extrathymic T cell development, along with differential B cell responses (Supplementary Information, Figure S4). Several efforts have been made to improve adaptive immune responses in humanized mouse models, such as delivering of recombinant cytokines as GM-CSF and IL-4 (ref. 41) or transgenic expression of HLA class I or II.^42,43^ SmyleDCpp65 may fulfill several of these requirements as it is fully HLA-matched with the transplanted HSC, expresses a wide array of cytokines and provides pp65 as a potent antigenic signal. Thus, besides the clinical value of SmyleDCpp65 for human HSCT, it also offers a practical tool for modeling human immune reconstitution in NRG mice in search for better immune therapeutic options.

Ultimately, although several viral and recombinant vaccine candidates against HCMV are currently being evaluated in clinical trials, immunization with SmyleDCpp65 is quite unique since it can accelerate thymic and extrathymic antigen-specific immune reconstitution in lymphopenic HSCT patients. Whereas the clinical development of SmyleDCpp65 from PB after leukapheresis is straightforward and ongoing (Sundarasetty *et al*, unpublished data), CB units have much lower number of monocytes. For the clinical development of SmyleDCpp65 as an adjuvant after CB-HSCT, a practical option would be to select the CD14^+^ cells from the whole CB unit used for transplantation or a fraction thereof, transduce with the lentiviral vector and cryopreserve. Cryopreservation/thawing did not alter the characteristics and properties of SmyleDCpp65 produced from PB or CB (Sundarasetty et al, unpublished data). Based on calculations of our results presented here (Supplementary Information, Table S1), starting with 800 million CB nucleated cells, we can recover ~40 million SmyleDCpp65. Taking into account that we may need half of these cells for quality control testing, we would have 20 million available for administration into patients. Clinical trials of DC immunotherapy have used typically 1–10^6^ cells for immunizations.

Incidentally, an additional potential use of SmyleDCpp65 would be to expand and activate T cells *in vitro* from HCMV negative donors or from cord blood or as was elegantly demonstrated.^30,44^ Since the multicistronic lentiviral system can be engineered for additional expression of transgenes or fusion proteins, antigenic proteins of adenovirus (ADV) and Epstein-Barr virus (EBV) could be coexpressed in SmyleDCpp65 in order to stimulate multiviral reactive T cells. To this end, simplified bulk cocultures of SmyleDCpp65/T cells with the purpose of obtaining large amounts of T cells in a short period of time for clinical use would have to be established. A current good manufacturing practices-compliant US FDA-approved manufacturing protocol of CB-derived *ex vivo* expanded T lymphocytes that target HCMV, EBV, and ADV takes more than 50 days.^45^ A large proportion of patients is infected or reactivate viral infections before day 35 after CB-HSCT. Using good manufacturing practices-compliant methods, we recently showed that cryopreserved SmyleDCpp65 from PB can be produced in a single day after leukapheresis. Quality control of the cells after thawing to determine identity (monocytes with detectable copies of ID-LV) and potency (immunophenotype, expression of antigen, and cytokine production) requires additional 10 days (Sundarasetty et al, unpublished data). Thus, the clinical logistics favors the use of SmyleDCpp65 directly as vaccine rather than expansion of T cells, but the two approaches could be also ideally combined.

In summary, SmyleDCpp65 is an innovative and feasible donor-derived cell vaccine to lower mortality and morbidity after PB-HSCT or CB-HSCT. Future additional pharmacodynamics, toxicology, and safety studies in humanized mouse models using SmyleDCpp65 produced under good manufacturing practices are now warranted to complement the preclinical potency studies shown here.

## Materials and Methods

Materials and methods are included as supplementary information and where indicated described elsewhere. Below are the methods and materials that were not described previously.

### Generation of lentivirus-vectored DCs with ID-LVs

PBMCs of HLA-A*02.01 /HLA-B*07.02 positive HCMV-reactive adult healthy volunteers, G-CSF (Granocyte, Chugai Pharma, London, UK) mobilized leukapheresis donors and umbilical CB were obtained in accordance with study protocols approved by Hannover Medical School Ethics Review Board. Generation of autologous SmyleDC from PBMCs and G-CSF mobilized blood obtained from adults was previously described.^13,16^ For generation of autologous SmyleDCpp65 from umbilical CB, PBMCs were initially used for CD34^+^ isolation and the negative CD34^-^ PBMC fraction was used for CD14^+^ monocytes isolation. Briefly, CD14^+^ was isolated using CD14 isolation beads (Miltenyi Biotech, Bergisch-Gladbach, Germany). The monocytes were preconditioned with recombinant human GM-CSF and IL-4 (50 ng/ml each, Cellgenix, Freiburg, Germany) in X-vivo-15 medium (Lonza, Cologne, Germany) for 8 hours prior to transduction. As a standard and upscalable protocol, 2.5 µg/ml p24 equivalent of ID-LV-pp65, ID-LV-G2α, or ID-LV-G2α-pp65 were used to transduce 5 × 10^6^ monocytes at multiplicity of infection of 5 in the presence of 5 µg/ml protamine sulfate (Valeant, Dusseldorf, Germany) for 16 hours. After transduction, the cells were washed twice with phosphate-buffered saline and further maintained in serum-free X-vivo-15 medium without additional cytokines for 7, 14, and 21 days *in vitro* for DC recovery, DC immunophenotype and cytokines analysis. For mice immunization, the transduced cells were resuspended in phosphate-buffered saline after washing and used directly for immunization. The number of viable counts was determined by trypan blue exclusion.

### HCMV-TB40/E GFP infection and plaque assay

Propagation of the HCMV TB40/E GFP strain^18^ on HF and preparation of virus stocks was propagated as previously described.^46^ Each type of target DCs was seeded at 5 × 10^5^ cells per well in six-well plates for each time point (0, 2, 4, 6, 8, and 10 days postinfection, d.p.i.). HF cells were used as a positive control. DCs and HF cells were infected with HCMV (at an multiplicity of infection of 1), washed extensively with phosphate-buffered saline after 24 hours, and further cultured with Dulbecco's modified Eagle's medium supplemented with 10% fetal bovine serum, and penicillin (100 U/ml) and streptomycin (100 µg/ml). Infected cells were harvested at each time point for GFP analysis and PE-conjugated anti-human CD80 was used for surface staining of DCs. After washing, cells were fixed in 1% paraformaldehyde and analyzed by flow cytometry. Supernatants of cell cultures were collected at the indicated time points and viral titers determined by standard plaque assays on HF^46^ using a carboxymethylcellulose overlay.

### Analyses of pp65-reactive T cells stimulated *in vitro*

Autologous CD3^+^ and CD8^+^ T cells were isolated from PBMCs of HLA-A*02.01/HLA-B*07.02 positive HCMV-reactive adult healthy volunteers using the MACS system following the manufacturer’s protocol (Miltenyi Biotec, Germany). For IFN-α intracellular staining analysis, T cells were stimulated for 16 h with 10 µg/ml PepTivator CMV-pp65 overlapping peptide pool (Miltenyi Biotec) or with DCs at ratio of 1:30 (APC:T-cell). Protein transport inhibitor cocktails (eBioscience, San Diego, CA) were added after 1 hour for 15 hours to inhibit protein transport. Stimulated T cells were harvested, stained with APC-conjugated anti-human CD3, PB-conjugated anti-human CD4, and PCy7-conjugated anti-human CD8 antibodies (Biolegend, San Diego, CA). After fixation/permeabilization with Cyofix/perm (BD Biosciences) for 20 minutes at 4 °C and washing, anti-human IFN-α (ebioscience) was used for staining for 30 minutes. The cells were analyzed by flow cytometry using LSRII (BD Biosciences, Heidelberg, Germany). For microculture T cell expansion assay, SmyleDC or SmyleDCpp65 (day 7) were cocultured with autologous isolated CD8^+^ T cells in 96-well plates at ratio of 1:10 (APC:T-cell) in X-vivo medium supplemented with 5% human AB serum. Gamma-irradiated autologous CD8^-^ feeder cells (2 × 10^5^) were added per microculture. After 3 days, the cells were replenished on alternate days with IL-2 (20 IU/ml) (Novartis Pharma GmbH, Nürnberg, Germany) IL-7 and IL-15 (5 ng/ml each, Cellgenix, Freiburg, Germany). For restimulation after 7 days expansion, cryopreserved DCs were thawed and added to T cells at 1:10 ratio. Re-stimulated T cells were harvested, counted and analyzed for pp65-reactivity by pentamer staining. PE-conjugated pentamer (HLA-A*0201-NLVPMVATV, pp65 amino acids (aa) 495–503; APC-HLA-B*0702-TPRVTGGGAM, pp65 aa 417–426; Proimmune, Oxford, UK), APC-conjugated anti-human CD3, PB-conjugated anti-human CD4, and PCy7-conjugated anti-human CD8 were used for staining.

### Analyses of thymus

Thymuses were harvested and single cell suspensions were subsequently stained with PB-conjugated anti-CD45, FITC-conjugated anti-CD3, A700-conjugated anti-CD4, APC-conjugated anti-CD3, FITC-conjugated anti-TCRαβ, and FITC-conjugated anti-TCR γδ followed by washing and analyzed by LSRII flow cytometry. Analyses of T cells at different stages of development in thymus DP; CD45^+^/CD4^+^/CD8^+^, CD4SP; CD45^+^/CD4^+^/CD8^-^, CD8SP; CD45^+^/CD4^-^/CD8^+^ CD3/TCRαβ; CD45^+^/CD3^hi^/TCRαβ^+^ analyses were performed using FlowJo (Tree Star, Ashland, OR) software. gDNA was extracted using the RNAzol bee (Ams Biotechnology, Frankfurt, Germany) according to the manufacturer’s instructions. Relative sjTREC levels were determined as previously described.^47^ PCRs were run in triplicate using Stratagene Mx3500P qPCR cycler (Agillient). Frequencies of sjTRECs were calculated by using ΔΔCt method. Frequency of TRECs in blood T cells from healthy donors (45–60 years) was set to 1 to calculate the relative frequency of TRECs in CB T cells and thymocytes from humanized recipients.

### Analyses of TCR-α and TCR-β chain repertoire

TCR sequencing for both α- and β-chain was performed in order to characterize the TCR repertoire diversity. RNA was isolated from total splenocytes isolated from control mice (*n* = 2) or mice immunized with SmyleDC/pp65 (*n* = 9) and SmyleDCpp65 (*n* = 2) cells using the RNAeasy purification kit (Qiagen, Hilden, Germany). PBMNC samples (*n* = 2) isolated from adult HSC donors that were used for mouse reconstitution were taken as control. cDNA was synthesized using the Superscript II reverse transcriptase (Invitrogen, Karlsruhe, Germany) and used for the analysis of the TCR diversity by an adapted nr/LAM PCR,^48,49^ independently of multiplex PCR reactions. The Illumina-specific sequencing adaptors containing 10 bp barcodes were added during the PCR. PCR amplicons were purified using the Agencourt Ampure beads (Beckman Coulter, Krefeld, Germany) and samples were sequenced using the MiSeq platform. After sequencing, raw reads were sorted according to the individual barcodes used for each sample and retrieval of the CDR3 α- and β-clonotypes was obtained by using the MiTCR software.^50^

### Statistical analysis

Nonparametric Man–Whitney *t*-test and analysis of variance with *post hoc* analysis tests were used for determining statistical significances. Data were analyzed with GraphPad Prism 5 (San Diego, CA) and SPSS software. * represents asymptotic significance analyzed by Kolmogorov–Smirnov test. All tests were two sided, and *P* < 0.05 was considered significant.

## References

[bib1] Boeckh M Ljungman P 2009 How we treat cytomegalovirus in hematopoietic cell transplant recipients Blood 113 5711 5719 19299333 10.1182/blood-2008-10-143560 PMC2700312

[bib2] Schmidt-Hieber M Labopin M Beelen D Volin L Ehninger G Finke J 2013 CMV serostatus still has an important prognostic impact in de novo acute leukemia patients after allogeneic stem cell transplantation: a report from the Acute Leukemia Working Party of EBMT Blood 122 3359 3364 24037724 10.1182/blood-2013-05-499830

[bib3] Boeckh M Nichols WG 2004 The impact of cytomegalovirus serostatus of donor and recipient before hematopoietic stem cell transplantation in the era of antiviral prophylaxis and preemptive therapy Blood 103 2003 2008 14644993 10.1182/blood-2003-10-3616

[bib4] Handgretinger R Klingebiel T Lang P Schumm M Neu S Geiselhart A 2001 Megadose transplantation of purified peripheral blood CD34(+) progenitor cells from HLA-mismatched parental donors in children Bone Marrow Transplant 27 777 783 11477433 10.1038/sj.bmt.1702996

[bib5] Aversa F Tabilio A Velardi A Cunningham I Terenzi A Falzetti F 1998 Treatment of high-risk acute leukemia with T-cell-depleted stem cells from related donors with one fully mismatched HLA haplotype N Engl J Med 339 1186 1193 9780338 10.1056/NEJM199810223391702

[bib6] Douek DC Vescio RA Betts MR Brenchley JM Hill BJ Zhang L 2000 Assessment of thymic output in adults after haematopoietic stem-cell transplantation and prediction of T-cell reconstitution Lancet 355 1875 1881 10866444 10.1016/S0140-6736(00)02293-5

[bib7] Milano F Pergam SA Xie H Leisenring WM Gutman JA Riffkin I 2011 Intensive strategy to prevent CMV disease in seropositive umbilical cord blood transplant recipients Blood 118 5689 5696 21937692 10.1182/blood-2011-06-361618 PMC3217367

[bib8] McGoldrick SM Bleakley ME Guerrero A Turtle CJ Yamamoto TN Pereira SE 2013 Cytomegalovirus-specific T cells are primed early after cord blood transplant but fail to control virus in vivo Blood 121 2796 2803 23412093 10.1182/blood-2012-09-453720 PMC3617639

[bib9] Steinman RM 2012 Decisions about dendritic cells: past, present, and future Annu Rev Immunol 30 1 22 22136168 10.1146/annurev-immunol-100311-102839

[bib10] Wendland M Willenzon S Kocks J Davalos-Misslitz AC Hammerschmidt SI Schumann K 2011 Lymph node T cell homeostasis relies on steady state homing of dendritic cells Immunity 35 945 957 22195748 10.1016/j.immuni.2011.10.017

[bib11] Verdijk P Aarntzen EH Lesterhuis WJ Boullart AC Kok E van Rossum MM 2009 Limited amounts of dendritic cells migrate into the T-cell area of lymph nodes but have high immune activating potential in melanoma patients Clin Cancer Res 15 2531 2540 19318472 10.1158/1078-0432.CCR-08-2729

[bib12] Grigoleit GU Kapp M Hebart H Fick K Beck R Jahn G 2007 Dendritic cell vaccination in allogeneic stem cell recipients: induction of human cytomegalovirus (HCMV)-specific cytotoxic T lymphocyte responses even in patients receiving a transplant from an HCMV-seronegative donor J Infect Dis 196 699 704 17674311 10.1086/520538

[bib13] Daenthanasanmak A Salguero G Borchers S Figueiredo C Jacobs R Sundarasetty BS 2012 Integrase-defective lentiviral vectors encoding cytokines induce differentiation of human dendritic cells and stimulate multivalent immune responses *in vitro* and in vivo Vaccine 30 5118 5131 22691433 10.1016/j.vaccine.2012.05.063

[bib14] Pearson T Shultz LD Miller D King M Laning J Fodor W 2008 Non-obese diabetic-recombination activating gene-1 (NOD-Rag1 null) interleukin (IL)-2 receptor common gamma chain (IL2r gamma null) null mice: a radioresistant model for human lymphohaematopoietic engraftment Clin Exp Immunol 154 270 284 18785974 10.1111/j.1365-2249.2008.03753.x PMC2612717

[bib15] Marodon G Desjardins D Mercey L Baillou C Parent P Manuel M 2009 High diversity of the immune repertoire in humanized NOD.SCID.gamma c-/- mice Eur J Immunol 39 2136 2145 19572320 10.1002/eji.200939480

[bib16] Salguero G Daenthanasanmak A Münz C Raykova A Guzmán CA Riese P 2014 Dendritic cell-mediated immune humanization of mice: implications for allogeneic and xenogeneic stem cell transplantation J Immunol 192 4636 4647 24740501 10.4049/jimmunol.1302887

[bib17] Riegler S Hebart H Einsele H Brossart P Jahn G Sinzger C 2000 Monocyte-derived dendritic cells are permissive to the complete replicative cycle of human cytomegalovirus J Gen Virol 81 Pt 2 393 399 10644837 10.1099/0022-1317-81-2-393

[bib18] Sinzger C Hahn G Digel M Katona R Sampaio KL Messerle M 2008 Cloning and sequencing of a highly productive, endotheliotropic virus strain derived from human cytomegalovirus TB40/E J Gen Virol 89 Pt 2 359 368 18198366 10.1099/vir.0.83286-0

[bib19] Moutaftsi M Mehl AM Borysiewicz LK Tabi Z 2002 Human cytomegalovirus inhibits maturation and impairs function of monocyte-derived dendritic cells Blood 99 2913 2921 11929782 10.1182/blood.v99.8.2913

[bib20] Riddell SR Greenberg PD 1995 Principles for adoptive T cell therapy of human viral diseases Annu Rev Immunol 13 545 586 7612234 10.1146/annurev.iy.13.040195.002553

[bib21] Einsele H Roosnek E Rufer N Sinzger C Riegler S Löffler J 2002 Infusion of cytomegalovirus (CMV)-specific T cells for the treatment of CMV infection not responding to antiviral chemotherapy Blood 99 3916 3922 12010789 10.1182/blood.v99.11.3916

[bib22] Kapp M Tan SM Einsele H Grigoleit G 2007 Adoptive immunotherapy of HCMV infection Cytotherapy 9 699 711 17917875 10.1080/14653240701656046

[bib23] Hervas-Stubbs S Perez-Gracia JL Rouzaut A Sanmamed MF Le Bon A Melero I 2011 Direct effects of type I interferons on cells of the immune system Clin Cancer Res 17 2619 2627 21372217 10.1158/1078-0432.CCR-10-1114

[bib24] Demeure CE Byun DG Yang LP Vezzio N Delespesse G 1996 CD31 (PECAM-1) is a differentiation antigen lost during human CD4 T-cell maturation into Th1 or Th2 effector cells Immunology 88 110 115 8707335 10.1046/j.1365-2567.1996.d01-652.x PMC1456463

[bib25] Kohler S Thiel A 2009 Life after the thymus: CD31+ and CD31- human naive CD4+ T-cell subsets Blood 113 769 774 18583570 10.1182/blood-2008-02-139154

[bib26] Sung H Schleiss MR 2010 Update on the current status of cytomegalovirus vaccines Expert Rev Vaccines 9 1303 1314 21087108 10.1586/erv.10.125 PMC3595507

[bib27] Griffiths PD Stanton A McCarrell E Smith C Osman M Harber M 2011 Cytomegalovirus glycoprotein-B vaccine with MF59 adjuvant in transplant recipients: a phase 2 randomised placebo-controlled trial Lancet 377 1256 1263 21481708 10.1016/S0140-6736(11)60136-0 PMC3075549

[bib28] Kharfan-Dabaja MA Boeckh M Wilck MB Langston AA Chu AH Wloch MK 2012 A novel therapeutic cytomegalovirus DNA vaccine in allogeneic haemopoietic stem-cell transplantation: a randomised, double-blind, placebo-controlled, phase 2 trial Lancet Infect Dis 12 290 299 22237175 10.1016/S1473-3099(11)70344-9

[bib29] Lilja AE Mason PW 2012 The next generation recombinant human cytomegalovirus vaccine candidates-beyond gB Vaccine 30 6980 6990 23041121 10.1016/j.vaccine.2012.09.056

[bib30] Hanley PJ Cruz CR Savoldo B Leen AM Stanojevic M Khalil M 2009 Functionally active virus-specific T cells that target CMV, adenovirus, and EBV can be expanded from naive T-cell populations in cord blood and will target a range of viral epitopes Blood 114 1958 1967 19443656 10.1182/blood-2009-03-213256 PMC2738578

[bib31] McLaughlin-Taylor E Pande H Forman SJ Tanamachi B Li CR Zaia JA 1994 Identification of the major late human cytomegalovirus matrix protein pp65 as a target antigen for CD8+ virus-specific cytotoxic T lymphocytes J Med Virol 43 103 110 8083644 10.1002/jmv.1890430119

[bib32] Moss P Khan N 2004 CD8(+) T-cell immunity to cytomegalovirus Hum Immunol 65 456 464 15172445 10.1016/j.humimm.2004.02.014

[bib33] Jahn G Scholl BC Traupe B Fleckenstein B 1987 The two major structural phosphoproteins (pp65 and pp150) of human cytomegalovirus and their antigenic properties J Gen Virol 68 (Pt 5) 1327 1337 3033138 10.1099/0022-1317-68-5-1327

[bib34] Varnum SM Streblow DN Monroe ME Smith P Auberry KJ Pasa-Tolic L 2004 Identification of proteins in human cytomegalovirus (HCMV) particles: the HCMV proteome J Virol 78 10960 10966 15452216 10.1128/JVI.78.20.10960-10966.2004 PMC521840

[bib35] Browne EP Shenk T 2003 Human cytomegalovirus UL83-coded pp65 virion protein inhibits antiviral gene expression in infected cells Proc Natl Acad Sci USA 100 11439 11444 12972646 10.1073/pnas.1534570100 PMC208776

[bib36] Abate DA Watanabe S Mocarski ES 2004 Major human cytomegalovirus structural protein pp65 (ppUL83) prevents interferon response factor 3 activation in the interferon response J Virol 78 10995 11006 15452220 10.1128/JVI.78.20.10995-11006.2004 PMC521853

[bib37] Cristea IM Moorman NJ Terhune SS Cuevas CD O’Keefe ES Rout MP 2010 Human cytomegalovirus pUL83 stimulates activity of the viral immediate-early promoter through its interaction with the cellular IFI16 protein J Virol 84 7803 7814 20504932 10.1128/JVI.00139-10 PMC2897612

[bib38] Williams KM Hakim FT Gress RE 2007 T cell immune reconstitution following lymphodepletion Semin Immunol 19 318 330 18023361 10.1016/j.smim.2007.10.004 PMC2180244

[bib39] McClory S Hughes T Freud AG Briercheck EL Martin C Trimboli AJ 2012 Evidence for a stepwise program of extrathymic T cell development within the human tonsil J Clin Invest 122 1403 1415 22378041 10.1172/JCI46125 PMC3314444

[bib40] Lang J Kelly M Freed BM McCarter MD Kedl RM Torres RM 2013 Studies of lymphocyte reconstitution in a humanized mouse model reveal a requirement of T cells for human B cell maturation J Immunol 190 2090 2101 23335750 10.4049/jimmunol.1202810 PMC3578183

[bib41] Chen Q He F Kwang J Chan JK Chen J 2012 GM-CSF and IL-4 stimulate antibody responses in humanized mice by promoting T, B, and dendritic cell maturation J Immunol 189 5223 5229 23089398 10.4049/jimmunol.1201789 PMC4106296

[bib42] Suzuki M Takahashi T Katano I Ito R Ito M Harigae H 2012 Induction of human humoral immune responses in a novel HLA-DR-expressing transgenic NOD/Shi-scid/γcnull mouse Int Immunol 24 243 252 22402880 10.1093/intimm/dxs045

[bib43] Jaiswal S Pazoles P Woda M Shultz LD Greiner DL Brehm MA 2012 Enhanced humoral and HLA-A2-restricted dengue virus-specific T-cell responses in humanized BLT NSG mice Immunology 136 334 343 22384859 10.1111/j.1365-2567.2012.03585.x PMC3385033

[bib44] Park KD Marti L Kurtzberg J Szabolcs P 2006 *In vitro* priming and expansion of cytomegalovirus-specific Th1 and Tc1 T cells from naive cord blood lymphocytes Blood 108 1770 1773 16675712 10.1182/blood-2005-10-006536 PMC1895512

[bib45] Hanley PJ Lam S Shpall EJ Bollard CM 2012 Expanding cytotoxic T lymphocytes from umbilical cord blood that target cytomegalovirus, Epstein-Barr virus, and adenovirus JoVEe3627 22588077 10.3791/3627 PMC3466930

[bib46] Borst EM Hahn G Koszinowski UH Messerle M 1999 Cloning of the human cytomegalovirus (HCMV) genome as an infectious bacterial artificial chromosome in Escherichia coli: a new approach for construction of HCMV mutants J Virol 73 8320 8329 10482582 10.1128/jvi.73.10.8320-8329.1999 PMC112849

[bib47] Hazenberg MD Otto SA Cohen Stuart JW Verschuren MC Borleffs JC Boucher CA 2000 Increased cell division but not thymic dysfunction rapidly affects the T-cell receptor excision circle content of the naive T cell population in HIV-1 infection Nat Med 6 1036 1042 10973325 10.1038/79549

[bib48] Schmidt M Schwarzwaelder K Bartholomae C Zaoui K Ball C Pilz I 2007 High-resolution insertion-site analysis by linear amplification-mediated PCR (LAM-PCR) Nat Methods 4 1051 1057 18049469 10.1038/nmeth1103

[bib49] Paruzynski A Arens A Gabriel R Bartholomae CC Scholz S Wang W 2010 Genome-wide high-throughput integrome analyses by nrLAM-PCR and next-generation sequencing Nat Protoc 5 1379 1395 20671722 10.1038/nprot.2010.87

[bib50] Bolotin DA Shugay M Mamedov IZ Putintseva EV Turchaninova MA Zvyagin IV 2013 MiTCR: software for T-cell receptor sequencing data analysis Nat Methods 10 813 814 23892897 10.1038/nmeth.2555

