# Supplementary figures and images for: Engineered dendritic cells from cord blood and adult blood accelerate effector T cell immune reconstitution against HCMV
Source: Mol Ther Methods Clin Dev. 2015 Jan 7;1:14060–. doi: 10.1038/mtm.2014.60 (PMC4449014; doi:10.1038/mtm.2014.60)

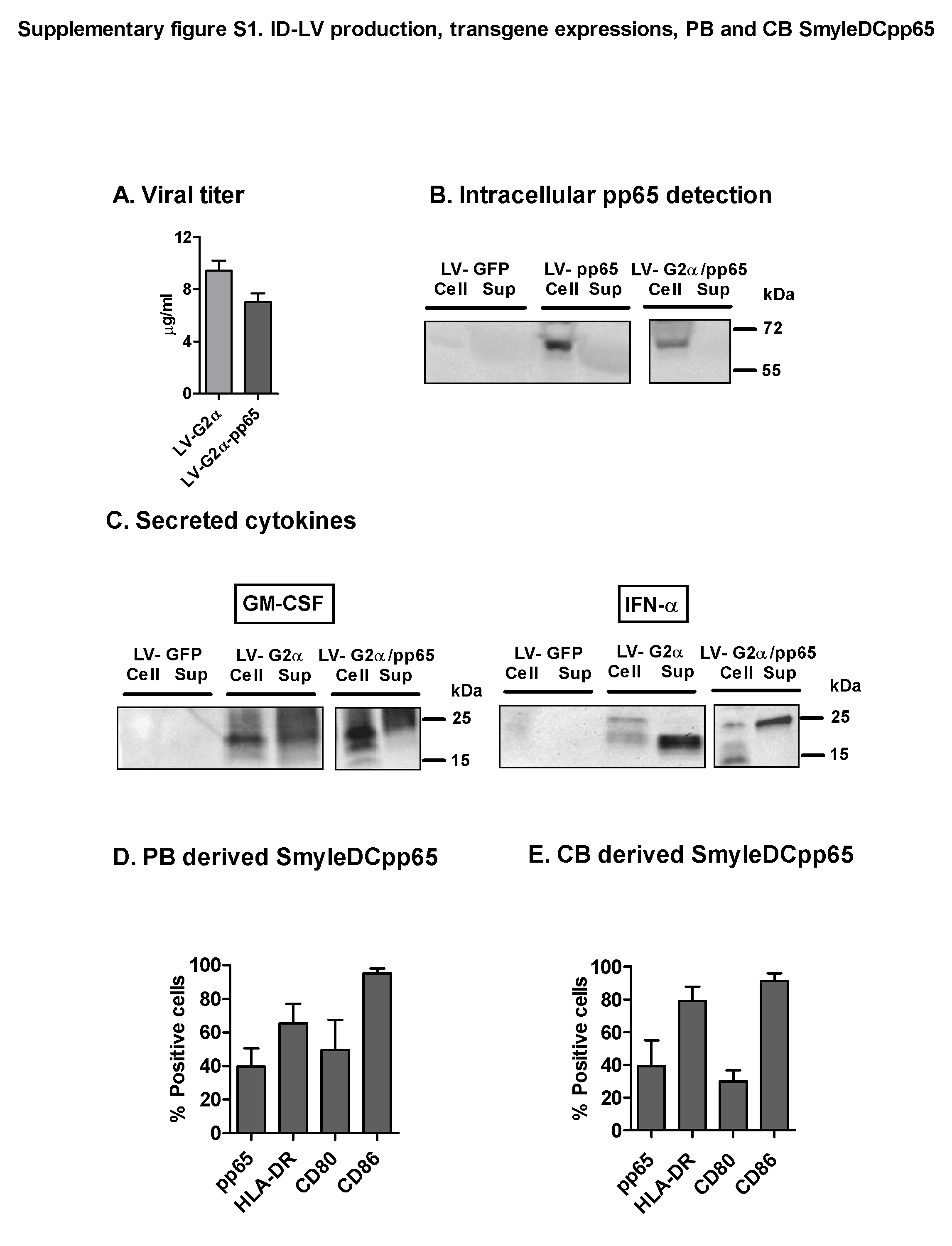

Supplement: Supplementary Figure S1 [file mtm201460-s1.tiff]

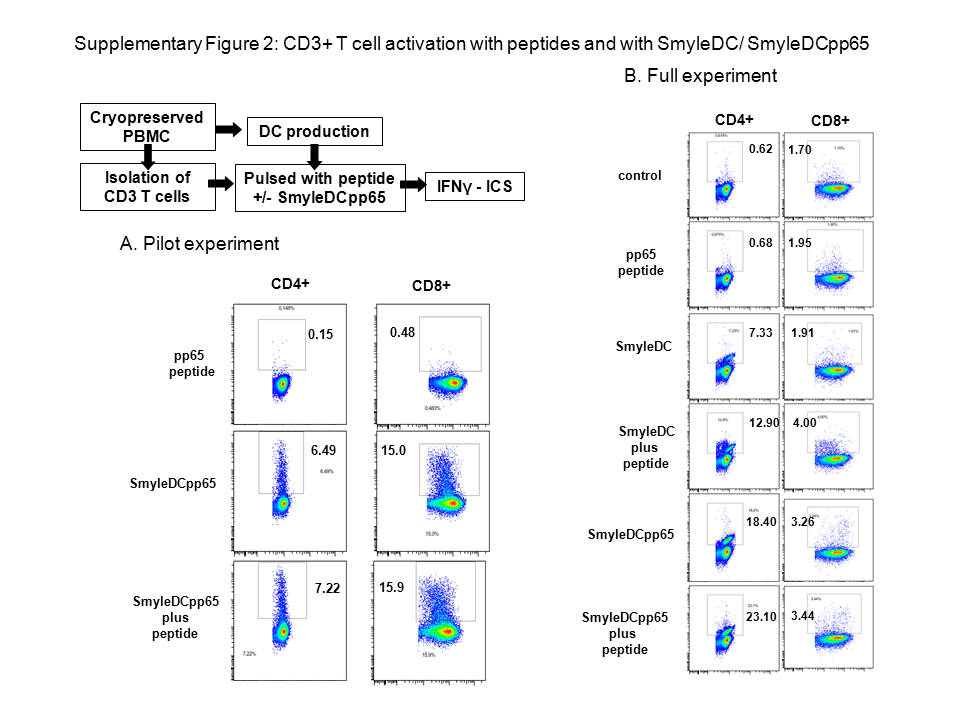

Supplement: Supplementary Figure S2 [file mtm201460-s2.tiff]

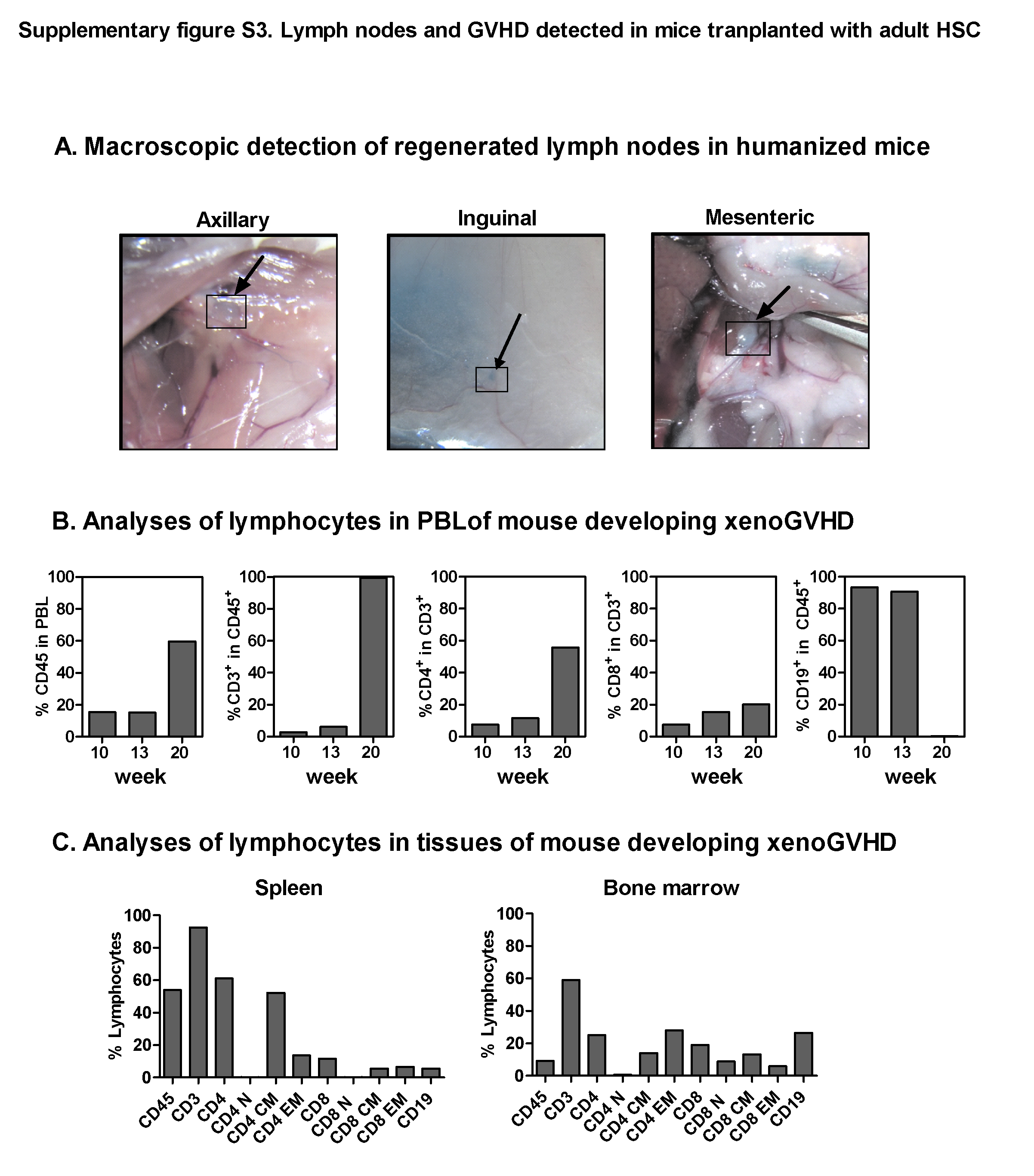

Supplement: Supplementary Figure S3 [file mtm201460-s3.tiff]

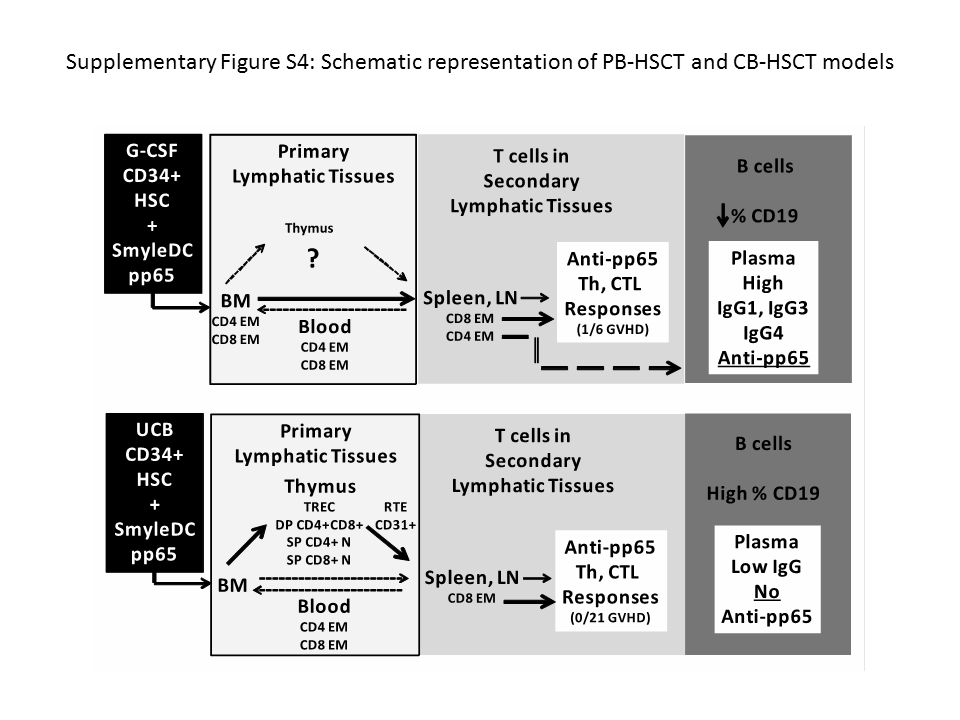

Supplement: Supplementary Figure S4 [file mtm201460-s4.tiff]
