# Supplementary material for: Engineered dendritic cells from cord blood and adult blood accelerate effector T cell immune reconstitution against HCMV
Source: Mol Ther Methods Clin Dev. 2015 Jan 7;1:14060–. doi: 10.1038/mtm.2014.60 (PMC4449014; doi:10.1038/mtm.2014.60)
Supplement: Supplementary Table S1 [file mtm201460-s5.doc]

**Supplementary Table 1. Generation of SmyleDCpp65 of cord blood monocytes.**

**After Ficoll / CD34 selection CD14+ selection Transduction LV-G2α-pp65**

| **#** | **Total PBMC** | **Vials frozen**  **(a´5x10^7^)** | **In** | **Out** | **Recovery** | **In** | **Out** | **Recovery** | **Overall Recovery** | **Projected # SmyleDCpp65 for CB Unit** |
| --- | --- | --- | --- | --- | --- | --- | --- | --- | --- | --- |
| 1 | 6,1x10^8^ | 10 | 5x10^7^ | 5,3x10^6^ | 0,108 | 5 x10^6^ | 1,9x10^6^ | 0,38 | 0,041 | 2,5 x10^7^ |
| 2 | 6,9x10^8^ | 11 | 5x10^7^ | 5,7 x10^6^ | 0,114 | 5 x10^6^ | 2,4x10^6^ | 0,48 | 0,055 | 3,8 x10^7^ |
| 3 | 9,0x10^8^ | 18 | 5x10^7^ | 5,9 x10^6^ | 0,118 | 5 x10^6^ | 2,5x10^6^ | 0,50 | 0,059 | 5,3 x10^7^ |

Average Recovery as Percentage 11,3 45,3 5,2 (3,8 x10^7^)
