# Supplementary material for: Engineered dendritic cells from cord blood and adult blood accelerate effector T cell immune reconstitution against HCMV
Source: Mol Ther Methods Clin Dev. 2015 Jan 7;1:14060–. doi: 10.1038/mtm.2014.60 (PMC4449014; doi:10.1038/mtm.2014.60)
Supplement: Supplementary Table S2 [file mtm201460-s6.doc]

**Supplementary Table 2. Stem cell sources used for stem cell transplantation in NRG mice**

| **Source** | **Donor ID** | **HLA-A** | **CMV status** | **Number of mice** |
| --- | --- | --- | --- | --- |
| **G-CSF mobilized blood** | BD001 | A*02, A*31 | IgG^+^ | 3 |
| **G-CSF mobilized blood** | BD006 | A*02, A*23 | IgG^+^ | 4 |
| **G-CSF mobilized blood** | BD005 | A*03, A*26 | IgG^+^ | 3 |
| **Umbilical cord blood** | CB13 | A*02 | N/A | 6 |
| **Umbilical cord blood** | CB14 | A*02 | N/A | 6 |
| **Umbilical cord blood** | CB22 | N/A | N/A | 5 |
| **Umbilical cord blood** | CB32 | A*02 | N/A | 2 |
| **Umbilical cord blood** | CB37 | A*02 | N/A | 14 |
