# Supplementary material for: Engineered dendritic cells from cord blood and adult blood accelerate effector T cell immune reconstitution against HCMV
Source: Mol Ther Methods Clin Dev. 2015 Jan 7;1:14060–. doi: 10.1038/mtm.2014.60 (PMC4449014; doi:10.1038/mtm.2014.60)
Supplement: Supplementary Table S3 [file mtm201460-s7.doc]

**Supplementary Table 3. Kolmogorov-Smirnov test for cytokine and immunoglobulin analysis**

**a.** Cytokine analysis (G-CSF mobilized model)

| **Source** | **IL-4** | **IL-12p70** | **IL-10** | **IL-5** | **IL-6** | **IL-8** | **IL-1β** | **TNF-α** | **IFN-γ** | **GM-CSF** | **MCP-1** |
| --- | --- | --- | --- | --- | --- | --- | --- | --- | --- | --- | --- |
| N | 9 | 9 | 9 | 9 | 9 | 9 | 9 | 9 | 9 | 9 | 9 |
| Parameter of average | 0.77 | 1.67 | 2.09 | 2.69 | 3.05 | 3.69 | 2.63 | 6.45 | 9.39 | 22.11 | 38.18 |
| Normal distribution of STD | 0.95 | 1.01 | 2.98 | 2.70 | 5.53 | 5.96 | 7.46 | 15.04 | 16.22 | 37.42 | 65.43 |
| Extreme differences in Absolute | 0.24 | 0.42 | 0.34 | 0.17 | 0.43 | 0.30 | 0.49 | 0.47 | 0.34 | 0.43 | 0.42 |
| Positive | 0.24 | 0.42 | 0.34 | 0.17 | 0.43 | 0.30 | 0.49 | 0.47 | 0.34 | 0.43 | 0.42 |
| Negative | -0.20 | -0.35 | -0.24 | -0.16 | -0.29 | -0.26 | -0.36 | -0.33 | -0.28 | -0.28 | -0.28 |
| Kolmogorov-Smirnov -Z | 0.72 | 1.35 | 1.04 | 0.52 | 1.29 | 0.90 | 1.49 | 1.40 | 1.03 | 1.31 | 1.28 |
| Asymptotic Significance (2-sided) | 0.67 | 0.05 | 0.22 | 0.94 | 0.07 | 0.38 | 0.23 | 0.03 | 0.23 | 0.06 | 0.07 |

**b.** Immunoglobulin analysis (G-CSF mobilized model)

| **Source** | **IgM** | **IgA** | **IgG1** | **IgG2** | **IgG3** | **IgG4** |
| --- | --- | --- | --- | --- | --- | --- |
| N | 10 | 10 | 10 | 10 | 10 | 10 |
| Parameter of average | 12,732.71 | 157.68 | 38,736.79 | 8,700.95 | 1,001.40 | 206 |
| Normal distribution of STD | 33,555.16 | 224,.47 | 82,056.82 | 19,365.95 | 2,917.37 | 590.01 |
| Extreme differences in Absolute | 0.42 | 0.30 | 0.47 | 0.39 | 0.44 | 0.43 |
| Positive | 0.42 | 0.42 | 0.47 | 0.39 | 0.44 | 0.43 |
| Negative | -0.35 | -0.35 | -0.31 | -0.32 | -0.36 | -0.36 |
| Kolmogorov-Smirnov -Z | 1.35 | 0.97 | 1.50 | 1.26 | 1.42 | 1.36 |
| Asymptotic Significance (2-sided) | 0.05 | 0.29 | 0.02 | 0.08 | 0.03 | 0.04 |
