# Supplementary material for: Engineered dendritic cells from cord blood and adult blood accelerate effector T cell immune reconstitution against HCMV
Source: Mol Ther Methods Clin Dev. 2015 Jan 7;1:14060–. doi: 10.1038/mtm.2014.60 (PMC4449014; doi:10.1038/mtm.2014.60)
Supplement: Supplementary Table S4 [file mtm201460-s8.docx]

**Supplementary Table 4. TCR analyses: α-chain statistics**

| **Group** | **Mouse ID #** | **Stem cell donor** | **Reconstitution #** | **Total Sequences** | **CDR3 (nt)** | **CDR3 (aa)** | **V genes** | **J genes** |
| --- | --- | --- | --- | --- | --- | --- | --- | --- |
| **PBMNC** | **-** | **BD001** | **-** | **56028** | **414** | **402** | **39** | **45** |
|  | **-** | **BD004** | **-** | **74676** | **618** | **603** | **40** | **47** |
| **Control** | **630** | **BD001** | **R6** | **8091** | **108** | **101** | **6** | **10** |
|  | **682** | **BD007** | **R13** | **1345** | **13** | **13** | **1** | **1** |
| **SmyleDC/**  **pp65**  **2 vectors*** | **698** | **BD001** | **R14** | **36334** | **353** | **299** | **31** | **35** |
|  | **702** | **BD001** | **R14** | **71414** | **436** | **408** | **35** | **44** |
|  | **703** | **BD001** | **R14** | **68104** | **847** | **828** | **43** | **48** |
|  | **696** | **BD001** | **R14** | **29976** | **145** | **134** | **20** | **25** |
|  | **693** | **BD007** | **R15** | **61459** | **272** | **249** | **25** | **28** |
|  | **695** | **BD007** | **R15** | **36932** | **230** | **209** | **27** | **30** |
|  | **1010** | **BD004** | **R8** | **87445** | **417** | **326** | **19** | **14** |
|  | **1011** | **BD004** | **R8** | **23927** | **105** | **99** | **7** | **7** |
| **SmyleDC**  **pp65**  **tricistronic** | **700** | **BD001** | **R14** | **34066** | **232** | **202** | **7** | **16** |
|  | **701** | **BD001** | **R14** | **80197** | **599** | **563** | **36** | **44** |

***RNA samples for TCR analyses were obtained from humanized mice described in Salguero et al, J. Immunology 2014**
