# Supplementary material for: Engineered dendritic cells from cord blood and adult blood accelerate effector T cell immune reconstitution against HCMV
Source: Mol Ther Methods Clin Dev. 2015 Jan 7;1:14060–. doi: 10.1038/mtm.2014.60 (PMC4449014; doi:10.1038/mtm.2014.60)
Supplement: Supplementary Table S5 [file mtm201460-s9.doc]

**Supplementary Table 5. TCR analyses: β**-chain statistics

| **Group** | **Mouse ID #** | **Stem cell donor** | **Reconstitution #** | **Total Sequences** | **CDR3 (nt)** | **CDR3 (aa)** | **V genes** | **J genes** |
| --- | --- | --- | --- | --- | --- | --- | --- | --- |
| **PBMNC** | **-** | **BD001** | **-** | **343** | **16** | **16** | **12** | **6** |
|  | **-** | **BD004** | **-** | **25083** | **698** | **695** | **43** | **13** |
| **Control** | **630** | **BD001** | **R6** | **993** | **12** | **12** | **5** | **5** |
|  | **682** | **BD007** | **R13** | **898** | **8** | **8** | **4** | **4** |
| **SmyleDC/**  **pp65**  **2 vectors*** | **698** | **BD001** | **R14** | **34888** | **447** | **437** | **42** | **13** |
|  | **702** | **BD001** | **R14** | **45518** | **443** | **423** | **38** | **13** |
|  | **703** | **BD001** | **R14** | **61984** | **910** | **883** | **45** | **13** |
|  | **696** | **BD001** | **R14** | **34023** | **303** | **298** | **32** | **12** |
|  | **693** | **BD007** | **R15** | **17264** | **131** | **127** | **14** | **8** |
|  | **695** | **BD007** | **R15** | **39099** | **334** | **322** | **40** | **12** |
|  | **1010** | **BD004** | **R8** | **7520** | **41** | **38** | **6** | **3** |
|  | **1011** | **BD004** | **R8** | **4556** | **33** | **33** | **11** | **8** |
| **SmyleDC**  **pp65**  **tricistronic** | **700** | **BD001** | **R14** | **10263** | **78** | **73** | **14** | **5** |
|  | **701** | **BD001** | **R14** | **16649** | **77** | **71** | **19** | **11** |

***RNA samples for TCR analyses were obtained from humanized mice described in Salguero et al, J. Immunology 2014**
